# LncRNA‐422 suppresses the proliferation and growth of colorectal cancer cells by targeting SFPQ

**DOI:** 10.1002/ctm2.664

**Published:** 2022-01-24

**Authors:** Yixuan Meng, Shuwei Li, Qiuyi Zhang, Shuai Ben, Qiuyuan Zhu, Mulong Du, Dongying Gu

**Affiliations:** ^1^ Department of Oncology Nanjing First Hospital Nanjing Medical University Nanjing China; ^2^ Department of Environmental Genomics Jiangsu Key Laboratory of Cancer Biomarkers, Prevention and Treatment Collaborative Innovation Center for Cancer Personalized Medicine Nanjing Medical University Nanjing China; ^3^ Department of Genetic Toxicology The Key Laboratory of Modern Toxicology of Ministry of Education Center for Global Health School of Public Health Nanjing Medical University Nanjing China; ^4^ Department of Biostatistics Center for Global Health School of Public Health Nanjing Medical University Nanjing China

In this study, the biological function of lncRNA‐422 was investigated in colorectal cancer, which suggested that down‐regulated lncRNA‐422 suppressed cell proliferation and inhibited tumor growth in cellular and xenograft models. Mechanistically, lncRNA‐422 could interact directly with splicing factor proline and glutamine rich (SFPQ) to activate downstream pathways. *SFPQ* drives cancer progression through diverse roles in RNA transcriptional activity, mRNA processing, splicing regulation and innate immune response in hepatocellular carcinoma,^1^ breast cancer,^2^ ovarian cancer^3^ and colorectal cancer.^4^ Moreover, *SFPQ* promoted the proliferation and onset of colorectal cancer.

LncRNA‐422 was previously identified^5^ and selected for further investigation in this study (Figure 1A). We found that lncRNA‐422 expression varied across multiple tissues but was enriched in colorectal tissues and cells (Figure 1B,C). LncRNA‐422 was significantly down‐regulated in colorectal cancer tissues from The Cancer Genome Atlas (TCGA), GSE104836, quantitative reverse transcription PCR (qRT‐PCR) and in‐house RNA‐Seq. Eighty‐six per cent (45 of 52) of the colorectal cancer tissues demonstrated down‐regulation of lncRNA‐422 expression compared to that in the normal tissues (Figure 1D‐H). Subsequent analysis showed that lncRNA‐422 expression was significantly correlated with ages <60 years (Table S1).

**FIGURE 1:**
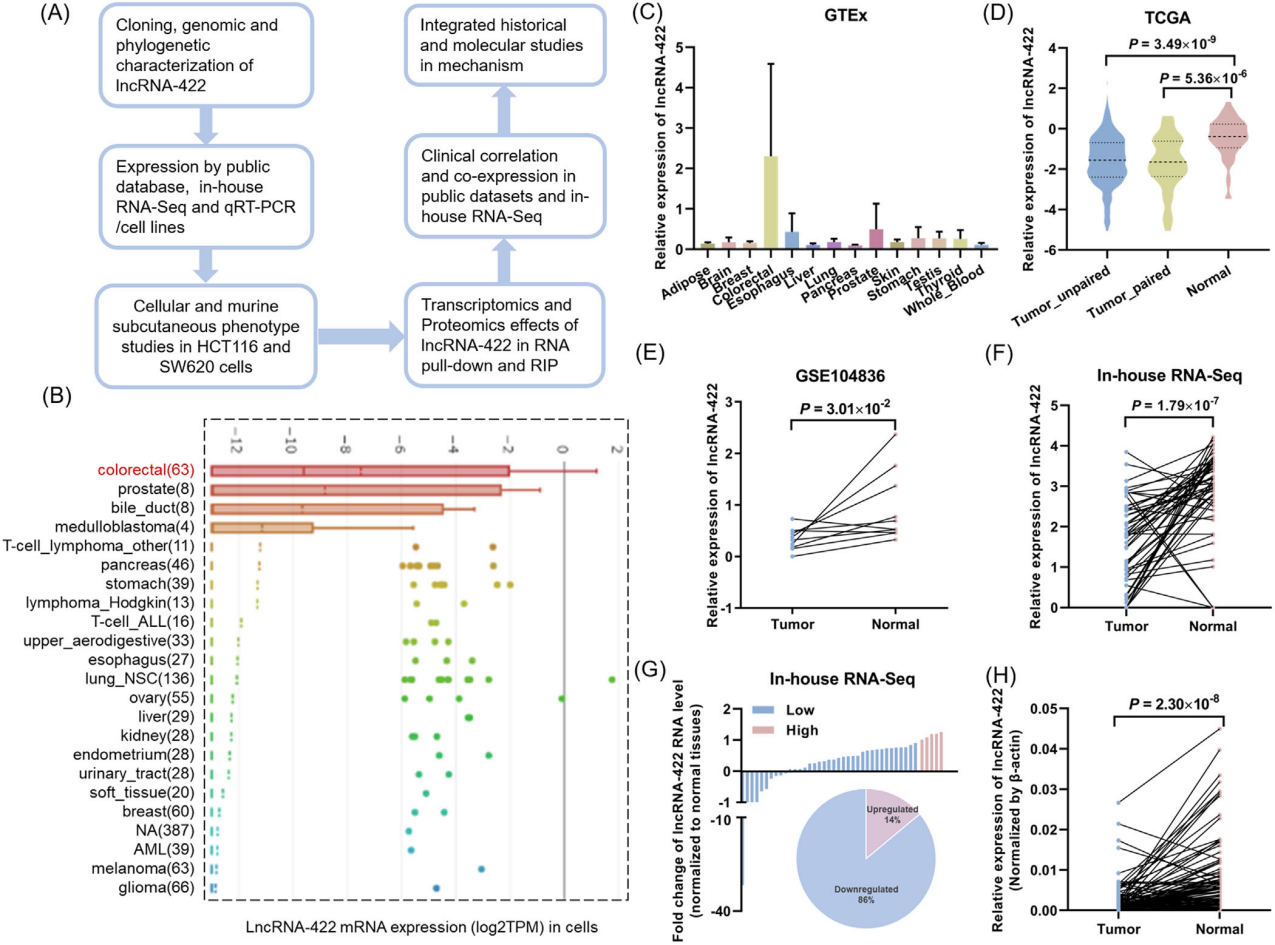
LncRNA‐422 expression is down‐regulated in colorectal cancer. (A) Schematic workflow of the identification and comprehensive functional characterization of lncRNA‐422 in colorectal carcinogenesis. LncRNA‐422 expression in Cancer Cell Line Encyclopedia (CCLE) (B), Genotype‐Tissue Expression Project (GTEx) (C), TCGA (D), GSE104836 (E) and 52 paired tissues (F). (G) Fold‐changes in lncRNA‐422 expression in 52 paired tissues (low, blue; high, pink). (H) LncRNA‐422 expression in colorectal cancer tissues compared with paired normal tissues detected by qRT‐PCR. All *P < 0.05 compared with the controls by a two‐sided Student's *t*‐test.

We evaluated the lncRNA‐422 transcript in primates with extremely high homology with the chimp and gorilla genomes (Figure S1A). In addition, we found that the coding potential of lncRNA‐422 for each transcript was negative, indicating that it is a noncoding RNA (Figure S1B‐E). Furthermore, full‐length lncRNA‐422 (855 bp in total) was identified on RNA isolated from HCT116 cells by using rapid amplification of cDNA ends assays (Figure S1F‐H). Taken together, the results support the lack of protein‐coding activity for lncRNA‐422.

Afterwards, we examined the biological significance of lncRNA‐422 down‐regulation in HCT116 and SW620 cells expressing low and high levels of lncRNA‐422, respectively (Figure S2A). Stable overexpression of lncRNA‐422 induced a significant decline in cellular growth, as verified by cell counting kit‐8 (CCK‐8) assay, 5‐Ethynyl‐2’‐deoxyuridine (EdU) assay and colony assay (Figure 2A‐C), increased the number of cells in S phase and induced apoptosis (Figure 2D‐E). Conversely, knocking down lncRNA‐422 significantly enhanced cellular functions (Figure S2B‐E). To confirm the observed phenotype,^6^ lncRNA‐422 xenograft tumors showed that lncRNA‐422 decreased the average volume, weight of tumors (Figure 2F‐H) and the expression of the proliferation marker β‐catenin (Figure 2I‐K). Together, these findings verified the tumor suppressor role of lncRNA‐422 in colorectal cancer.

**FIGURE 2:**
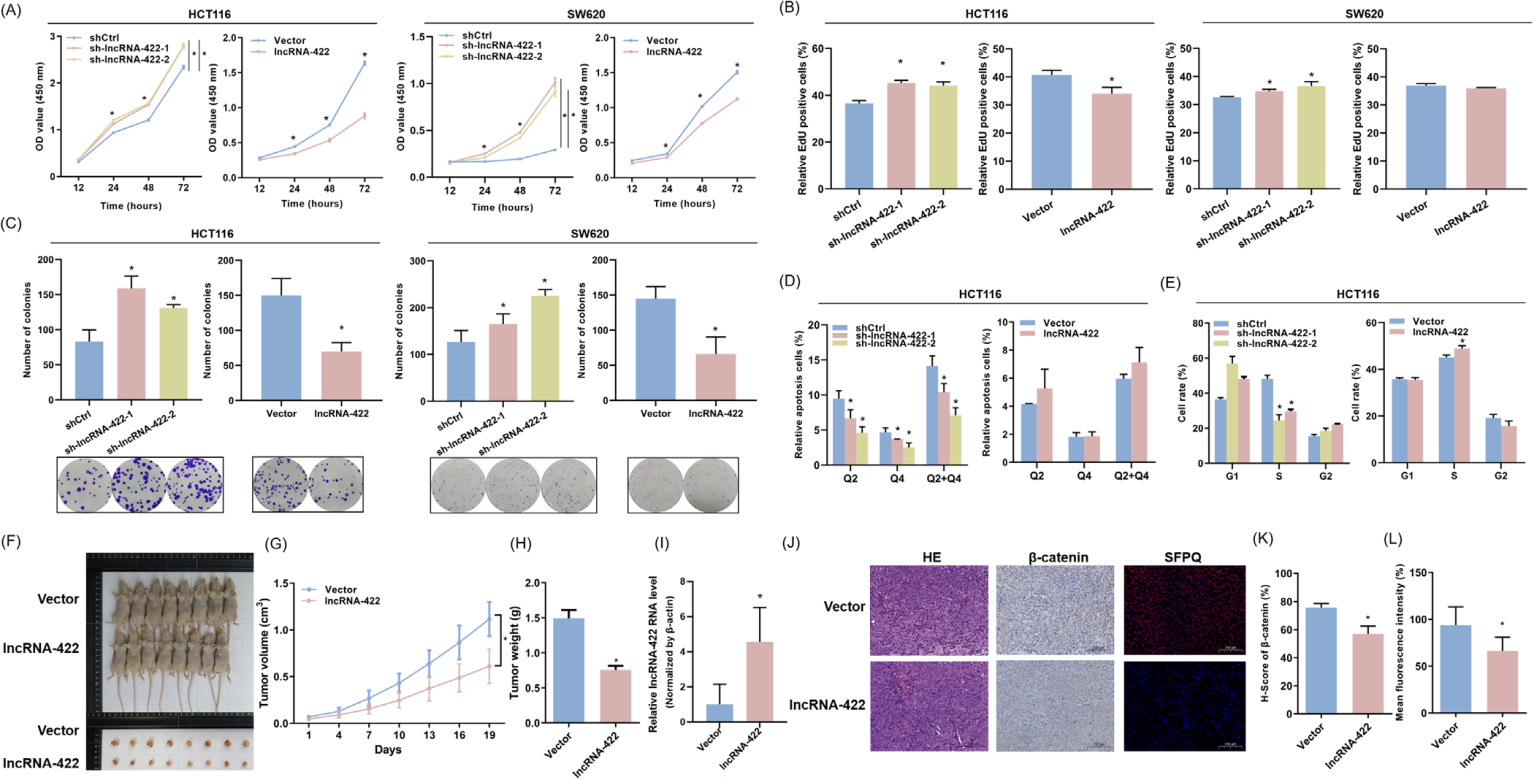
LncRNA‐422 inhibits the proliferation and increases apoptosis of colorectal cancer in cellular and murine subcutaneous models. Two independent shRNAs for lncRNA‐422 (sh‐lncRNA‐422‐1 and sh‐lncRNA‐422‐2), control shRNA (shCtrl), lncRNA‐422 stable overexpression vector (lncRNA‐422) or NC vectors (Vector) were transfected into HCT116 and SW620 cells. (A) Cell viability of lncRNA‐422 was evaluated by the cell counting kit‐8 (CCK‐8) assay. (B) The prominent effects of lncRNA‐422 on proliferation were confirmed using an EdU incorporation assay. (C) Colony formation was measured under the indicated lncRNA‐422 transfection conditions. (D) Flow cytometry analysis of apoptotic HCT116 cells. (E) The effect of HCT116 cells on the cell cycle process. (F) Nude mice were subcutaneously injected with the indicated HCT116 cell lines. (G) Tumor volume was measured in the lncRNA‐422 and negative control groups. (H) Tumor weight was measured in the lncRNA‐422 and negative control groups. (I) LncRNA‐422 expression was measured in xenograft colorectal cancer models. (J) Hematoxylin‐eosin staining (HE) staining along with immunohistochemistry (IHC) and Immunofluorescence (IF) showed the levels of SFPQ and the proliferation marker β‐catenin. Scale bars = 100 μm. (K) H‐score of β‐catenin in the xenograft colorectal cancer models. (L) Fluorescence intensity of SFPQ in xenograft colorectal cancer models. The flow cytometry images are representative of three repeated experiments. Data are shown as the mean ± SD. All *P < 0.05 compared with the controls by a two‐sided Student's *t*‐test.

We first identified that lncRNA‐422 is predominantly localized in the cell nucleus^7^ (Figure 3A,B). We then used unbiased approaches to identify intracellular lncRNA‐422‐interacting proteins,^8^ including RNA pull‐down, silver staining and mass spectrometry analysis^9^ (Figure 3C). Three potential interacting proteins were identified based on peptide number >2 and sequence coverage number >10 in the sense group and were absent in the antisense group (Table S2). Furthermore, independent western blotting confirmed that sense but not antisense lncRNA‐422 was significantly bound to SFPQ (Figure 3D).^10^ Importantly, RIP‐qPCR indicated that immunoprecipitation with an anti‐SFPQ antibody specifically retrieved lncRNA‐422 (Figure 3E). We confirmed that lncRNA‐422 can directly affect the protein level of SFPQ (Figure 3F). Collectively, these results suggest that lncRNA‐422 physically interacts with SFPQ in colorectal cancer cells.

**FIGURE 3:**
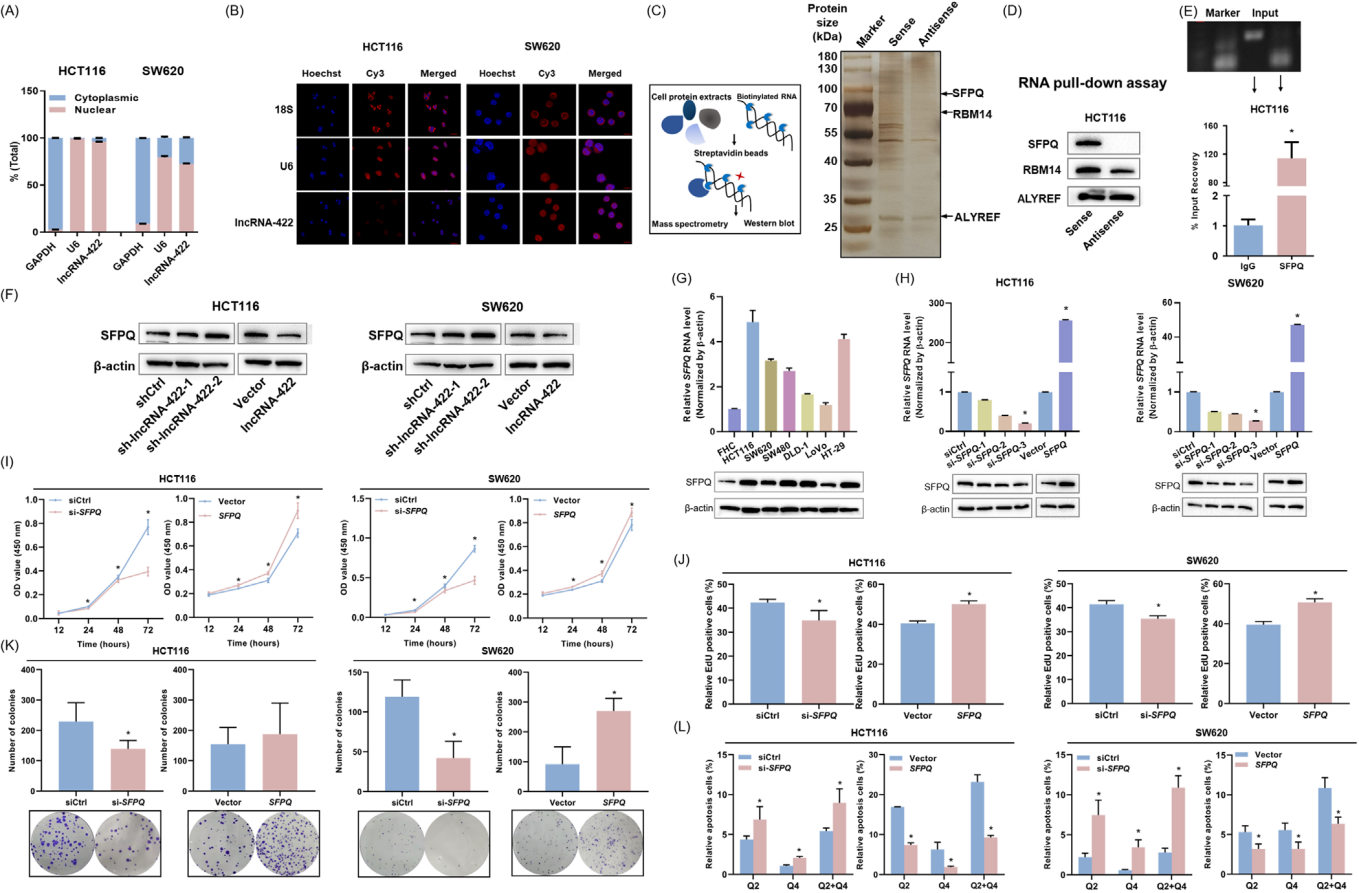
LncRNA‐422 interacts and regulates *SFPQ* expression in colorectal cancer cells. *SFPQ* siRNA (si‐*SFPQ*), control siRNA (siCtrl), *SFPQ* expression plasmid (*SFPQ*) or NC vector (Vector) was transfected into HCT116 and SW620 cells. (A) Subcellular fractionation of lncRNA‐422 in colorectal cancer cell lines. (B) LncRNA‐422 was mainly localized in the nucleus, as confirmed by fluorescence in situ hybridization (FISH) staining in colorectal cancer cell lines. Scale bar: 25 μm. Nuclei were stained blue (4′,6‐diamidino‐2‐phenylindole, DAPI), and the three RNAs (18S, U6 and lncRNA‐422) were stained red (Cy3). The 18S and U6 RNAs served as internal controls and were mainly located in the cytoplasm and nucleus, respectively. (C) Mechanism diagram of RNA pull‐down assays. lncRNA‐422‐sense and lncRNA‐422‐antisense RNAs were biotinylated, transcribed in vitro and incubated with HCT116 total cell lysates for RNA pull‐down assays. After silver staining, lncRNA‐422‐sense‐specific bands were excised and analysed using mass spectrometry. (D) Western blotting for the specific associations of SFPQ, RBM14 or ALYREF with biotinylated lncRNA‐422 from streptavidin RNA pull‐down assays. (E) RNA‐binding protein immunoprecipitation (RIP) assay was performed using the indicated SFPQ antibody. (F) The relative expression of *SFPQ* was determined in colorectal cancer cells after lncRNA‐422 transfection by western blotting. (G) *SFPQ* expression in different colorectal cancer cell lines (FHC, HCT116, SW620, SW480, DLD‐1, LoVo and HT‐29) was detected by qRT‐PCR and western blotting. (H) The relative expression of *SFPQ* was determined in colorectal cancer cells by qRT‐PCR and western blotting. (I) Cell viability was evaluated by the cell counting kit‐8 (CCK‐8) assay after *SFPQ* transfection. (J) The prominent effects of *SFPQ* on proliferation were confirmed using an EdU incorporation assay. (K) Colony formation was measured after *SFPQ* knockdown and overexpression. (L) Flow cytometry analysis of apoptotic colorectal cancer cells transfected with *SFPQ*. Flow cytometry images are representative of three repeated experiments. Data are shown as the mean ± SD from three repeated experiments, each with three replicates. All *P < 0.05 compared with the controls by a two‐sided Student's *t*‐test.

Specific small interfering RNA (siRNA) for *SFPQ* remarkably invoked proliferation defects, inhibited colony formation and induced apoptosis abilities, while *SFPQ* overexpression significantly increased the cellular functions in two colorectal cancer cells (Figure 3G‐L, Figure S3), validating the tumor‐promoting role of *SFPQ*. Furthermore, silencing *SFPQ*/lncRNA‐422 enhanced the expression level of lncRNA‐422/*SFPQ* (Figure S4), suggesting *SFPQ* as a potential mediator of lncRNA‐422 regulatory functions.

*SFPQ* knockdown mimicked the influence of lncRNA‐422, whereas *SFPQ* overexpression prevented lncRNA‐422‐induced proliferation and colony formation, and *SFPQ* knockdown restored the cell apoptosis effects reduced by lentivirus‐based knockdown of lncRNA‐422 (Figure 4A‐D, Figure S5). Together, these results suggest that lncRNA‐422 exerts tumor‐suppressing effects at least partially by binding to and inhibiting *SFPQ* activity in colorectal cancer.

**FIGURE 4:**
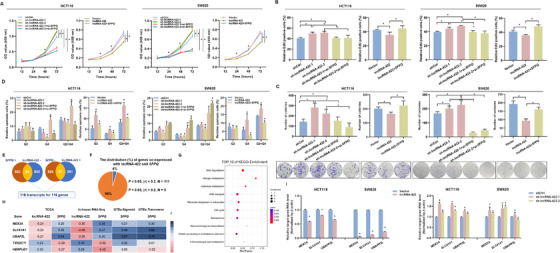
*SFPQ* mediates lncRNA‐422‐driven colorectal cancer cell growth, cell cycle progression and apoptosis activates a panel of genes. Two independent shRNAs for lncRNA‐422 (sh‐lncRNA‐422‐1 and sh‐lncRNA‐422‐2), control shRNA (shCtrl), lncRNA‐422 stable overexpression vector (lncRNA‐422) or NC vectors (Vector) were transfected into HCT116 and SW620 cells, and then *SFPQ* expression plasmid or si‐*SFPQ* was transfected into lncRNA‐422 or shRNAs cells, generating lncRNA‐422 + *SFPQ*, sh‐lncRNA‐422‐1 + si‐*SFPQ*, sh‐lncRNA‐422‐2 + si‐*SFPQ* cells. (A) The viability of colorectal cancer cells was evaluated by the cell counting kit‐8 (CCK‐8) assay. (B) The prominent effects of lncRNA‐422 and *SFPQ* constructs on proliferation were confirmed using an EdU incorporation assay. (C) Colony formation was measured under the indicated lncRNA‐422 and *SFPQ* transfection conditions. (D) Flow cytometry analysis of apoptotic colorectal cancer cells transfected with lncRNA‐422 and *SFPQ* constructs. (E) Venn diagram from TCGA and in‐house RNA‐Seq data analysis for colorectal cancer tissues depicting the differentially expressed genes regulated by lncRNA‐422 and *SFPQ*. (F) The distribution of genes co‐expressed with lncRNA‐422 and *SFPQ*. Among them, five candidate genes met the criterion: |*r*| > 0.2, *P* < 0.05. The values *P* < 0.05 and |*r*| > 0.2 were selected as the threshold of significance and calculated by Pearson's correlation analysis. (G) Pathway enrichment analysis of 116 target genes revealed that the majority of these genes are involved in multiple metabolic pathways. (H) The correlations of lncRNA‐422 and *SFPQ* expression with the expression of five candidate genes were evaluated by the TCGA, in‐house RNA‐Seq and GTEx dataset of sigmoid and transverse samples. The red and blue marks on the diagram indicate negative or positive correlations of *r*, respectively. (I) Relative expression of three target genes under the indicated lncRNA‐422 transfection conditions in colorectal cancer cells. Data are shown as the mean ± SD from three repeated experiments, each with three replicates. All *P < 0.05 compared with the controls by a two‐sided Student's *t*‐test.

We assessed the clinical significance of *SFPQ* in TCGA, GEO, in‐house RNA‐Seq and combined datasets (Figure S6A‐G). Remarkably, we first assessed the protein level of SFPQ in 30 paired in‐house microarrays using IHC and found that more than half of colorectal cancer tissues showed increased SFPQ expression compared with that in paired normal tissues (Figure S6H). Strong correlations were also identified in colorectal cancer tissues (*r* = ‐0.16, *P* = 3.00 × 10^–4^; Figure S6I). Intriguingly, the age at colorectal cancer diagnosis was decreased even further in patients with high *SFPQ* and low lncRNA‐422 expression (Figure S6J,K). Together, these integrated analyses indicate that the effects of *SFPQ* and lncRNA‐422 lead to colorectal cancer at a younger age.

We then tested the co‐expression of *SFPQ* and lncRNA‐422 in the TCGA cohort and in‐house RNA‐Seq on colorectal cancer tissues (*P *< 0.05, Figure 4E,F, Table S3). Further Kyoto Encyclopedia of Genes and Genomes analysis found that a total of 116 genes were enriched in RNA degradation and other metabolic pathways (Figure 4G, Figure S7). We validated the co‐expression of the candidate genes in the Genotype‐Tissue Expression (GTEx) databases (*r *> 0.20, *P *< 0.05, Figure 4H) and targeted three genes, *MEX3A*, *SLC41A1* and *UBAP2L*, which was confirmed with qRT‐PCR (Figure 4I).

In conclusion, this study revealed a novel lncRNA, lncRNA‐422, that regulates tumor cell proliferation and growth by targeting *SFPQ*. We focused on exploring the histological, molecular and cellular functions of lncRNA‐422 and discovered that lncRNA‐422 accelerates proliferation and tumor growth in cells and nude mouse models. RNA pull‐down, mass spectrometry and RIP assay demonstrated that lncRNA‐422 directly binds to SFPQ, leading to the change of malignancy. The lack of our mutation data and cells limits further validation, while we consider *SFPQ* mutants to affect interaction. LncRNA‐422 and *SFPQ* knockdown shared highly similar patterns in terms of cell proliferation and apoptosis, which supports the combined evaluation of lncRNA‐422 and *SFPQ* may be an efficient indicator for colorectal cancer prognosis. These results implicate lncRNA‐422/*SFPQ* as a novel target in colorectal cancer development and provide a basic understanding of lncRNA‐targeted molecular therapy.

## CONFLICT OF INTEREST

The authors declare that they have no conflict of interest.

## Supporting information

Supporting Information
