# Supplementary material for: LncRNA‐422 suppresses the proliferation and growth of colorectal cancer cells by targeting SFPQ
Source: Clin Transl Med. 2022 Jan 24;12(1):e664. doi: 10.1002/ctm2.664 (PMC8787101; doi:10.1002/ctm2.664)
Supplement: Supplementary file 1 — Supporting Information [file CTM2-12-e664-s001.docx]

**Figure legends**

**Figure S1. Conservation and characterization of the protein coding potential of lncRNA-422 in colorectal cancer.** (A) Graphical views showing multispecies comparisons of lncRNA-422 using the UCSC genome browser. The protein coding potential of lncRNA-422 was determined using ORF finder software from the National Center for Biotechnology Information (NCBI, https://www.ncbi.nlm.nih.gov/orffinder/) (B), PhyloCSF software (<http://compbio.mit.edu/PhyloCSF>) (C), Coding Potential Assessment Tool (CPAT, <http://lilab.research.bcm.edu/cpat/>) (D), and Coding Potential Calculator (CPC, http://cpc.cbi.pku.edu.cn/) (E). GAPDH and ACTB served as the positive controls for coding genes, and UCA1 and HOTAIR served as the negative controls for non-coding genes. (F) Representative images of PCR products from 5’ RACE and 3’ RACE. (G) Sequence of lncRNA-422 cloned from HCT116 cell cDNA. The sequences identified by RACE assays are underlined. (H) Sequencing of RACE products is shown from the 5’ RACE and 3’ RACE. The red arrows indicate the major PCR products.

**Figure S2.** **LncRNA-422 induces cell apoptosis but not the cell cycle.** Two independent shRNAs for lncRNA-422 (sh-lncRNA-422-1 and sh-lncRNA-422-2), control shRNA (shCtrl), lncRNA-422 stable overexpression vector (lncRNA-422) or NC vectors (Vector) were transfected into HCT116 and SW620 cells. (A) LncRNA-422 expression in colorectal cancer cell lines (HCT116, SW620, SW480, DLD-1, HT-29 and LoVo) was detected by qRT-PCR. (B) Relative expression levels of lncRNA-422 were determined by qRT–PCR in HCT116 and SW620 cells. (C) The prominent effects of lncRNA-422 on proliferation were confirmed using an EdU incorporation assay. (D) Flow cytometry analysis of apoptotic colorectal cancer cells. (E) The effect of colorectal cancer cells on the cell cycle process. Flow cytometry images are representative of three repeated experiments. Data are shown as the mean ± SD from three repeated experiments, each with three duplicates. All **P* < 0.05 compared with the controls by a two-sided Student’s *t*-test.

**Figure S3.** ***SFPQ* inhibits cell apoptosis but not the cell cycle.** *SFPQ* siRNA (si-*SFPQ)*, control siRNA (siCtrl), *SFPQ* expression plasmid (*SFPQ)* or NC vector (Vector) was transfected into HCT116 and SW620 cells. (A) The prominent effects of *SFPQ* on proliferation were confirmed using an EdU incorporation assay. (B) Effect of *SFPQ* on the apoptosis of colorectal cancer cells. (C) Effect of *SFPQ* on the cell cycle progression of colorectal cancer cells. Flow cytometry images are representative of three repeated experiments. Data are shown as the mean ± SD from three repeated experiments, each with three duplicates. All **P* < 0.05 compared with the controls by a two-sided Student’s *t*-test.

**Figure S4. Relative expression of lncRNA-422 and *SFPQ* in colorectal cancer cell lines.** Two independent shRNAs for lncRNA-422 (sh-lncRNA-422-1 and sh-lncRNA-422-2), control shRNA (shCtrl), lncRNA-422 stable overexpression vector (lncRNA-422) or NC vectors (Vector) were transfected into HCT116 and SW620 cells, and then *SFPQ* expression plasmid or si-*SFPQ* was transfected into lncRNA-422 or shRNAs cells, generating lncRNA-422 + *SFPQ*, sh-lncRNA-422-1 + si-*SFPQ*, sh-lncRNA-422-2 + si-*SFPQ* cells. (A) Relative expression levels of *SFPQ* were determined by qRT–PCR in colorectal cancer cells. (B) Relative expression levels of lncRNA-422 were determined by qRT–PCR in colorectal cancer cells. Data are shown as the mean ± SD from three repeated experiments, each with three duplicates. All **P* < 0.05 compared with the controls by a two-sided Student’s *t*-test.

**Figure S5.** ***SFPQ* mediates lncRNA-422-driven apoptosis and cell cycle effects in colorectal cancer cells.** Two independent shRNAs for lncRNA-422 (sh-lncRNA-422-1 and sh-lncRNA-422-2), control shRNA (shCtrl), lncRNA-422 stable overexpression vector (lncRNA-422) or NC vectors (Vector) were transfected into HCT116 and SW620 cells, and then *SFPQ* expression plasmid or si-*SFPQ* was transfected into lncRNA-422 or shRNAs cells, generating, lncRNA-422 + *SFPQ,* sh-lncRNA-422-1 + si-*SFPQ*, sh-lncRNA-422-2 + si-*SFPQ* cells. (A) The prominent effects of *SFPQ* and lncRNA-422 constructs on proliferation were confirmed using an EdU incorporation assay. (B) Effect of *SFPQ* and lncRNA-422 constructs on the apoptosis of colorectal cancer cells. (C) Effect of *SFPQ* and lncRNA-422 constructs on the cell cycle progression of colorectal cancer cells. Data are shown as the mean ± SD from three repeated experiments, each with three duplicates. All **P* < 0.05 compared with the controls by a two-sided Student’s *t*-test.

**Figure S6. Highly expressed *SFPQ* is associated with the synergy of lncRNA-422 in colorectal cancer tissues.** *SFPQ* expression values across colorectal cancer with paired and unpaired normal tissues in TCGA (A), GSE15781 (B), GSE8671 (C), GSE9348 (D), GSE21510 (E) and in-house RNA-Seq (F). (G) Forest plot of the meta-analyses of the associations between *SFPQ* and colorectal cancer risk in the six studies. The horizontal axis depicts the SMD and 95% CI. (H) The H-score of SFPQ of colorectal cancer and normal tissues in the in-house microarray. (I) The correlation between *SFPQ* and lncRNA-422 expression in TCGA colorectal cancer tissues. The synergy of *SFPQ* (J) and lncRNA-422 (K) significantly facilitates colorectal cancer at a younger age in TCGA colorectal cancer patient sets.

**Figure S7. Hierarchical cluster heatmap of differentially expressed genes in colorectal cancer and corresponding normal tissues generated from the TCGA database.** Red in the heatmap denotes upregulation; blue denotes downregulation.

**Figure S8. Original images of western blot.**

**Figure S1**

**
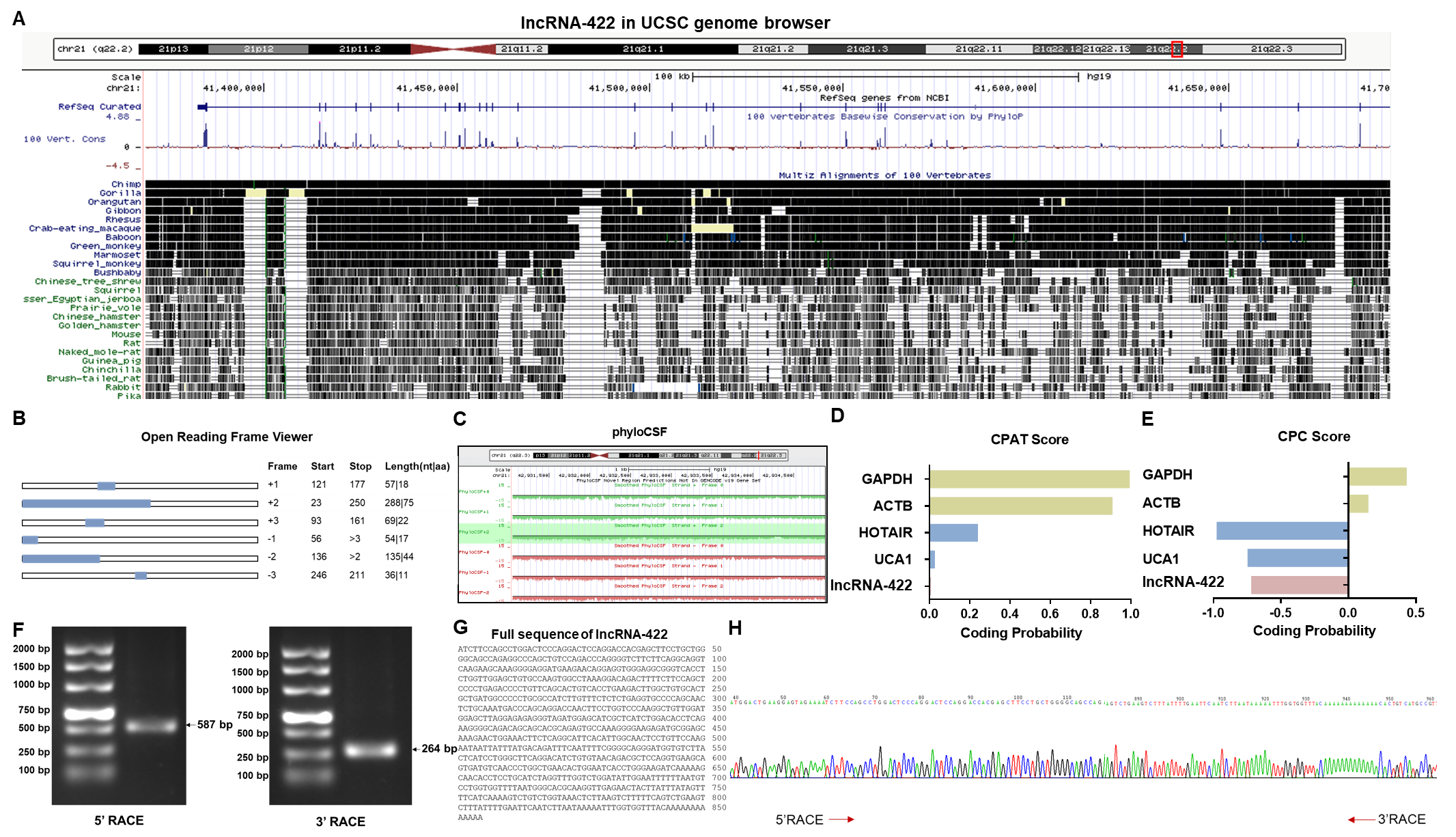
**

**Figure S2**

**
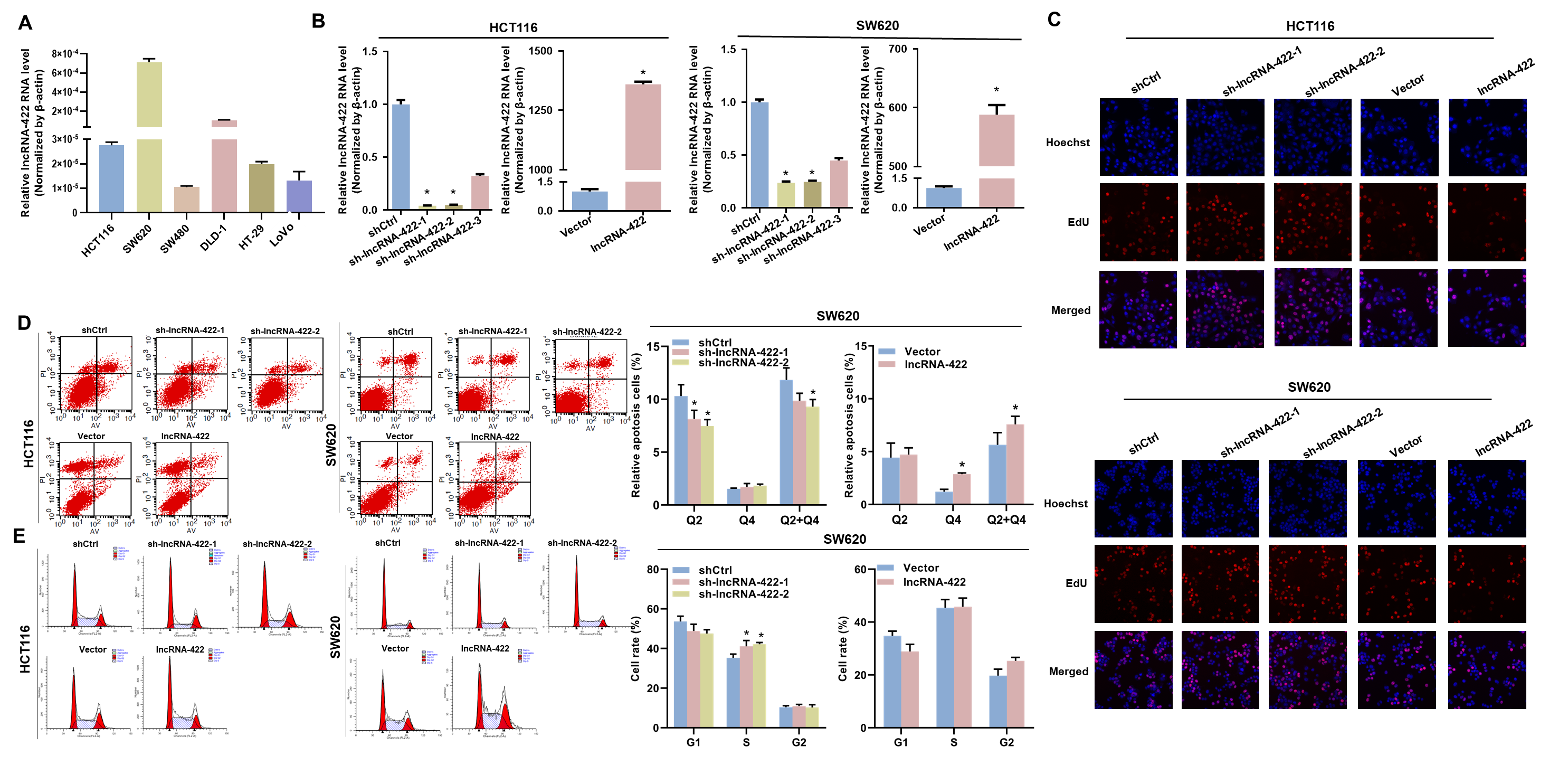
**

**Figure S3
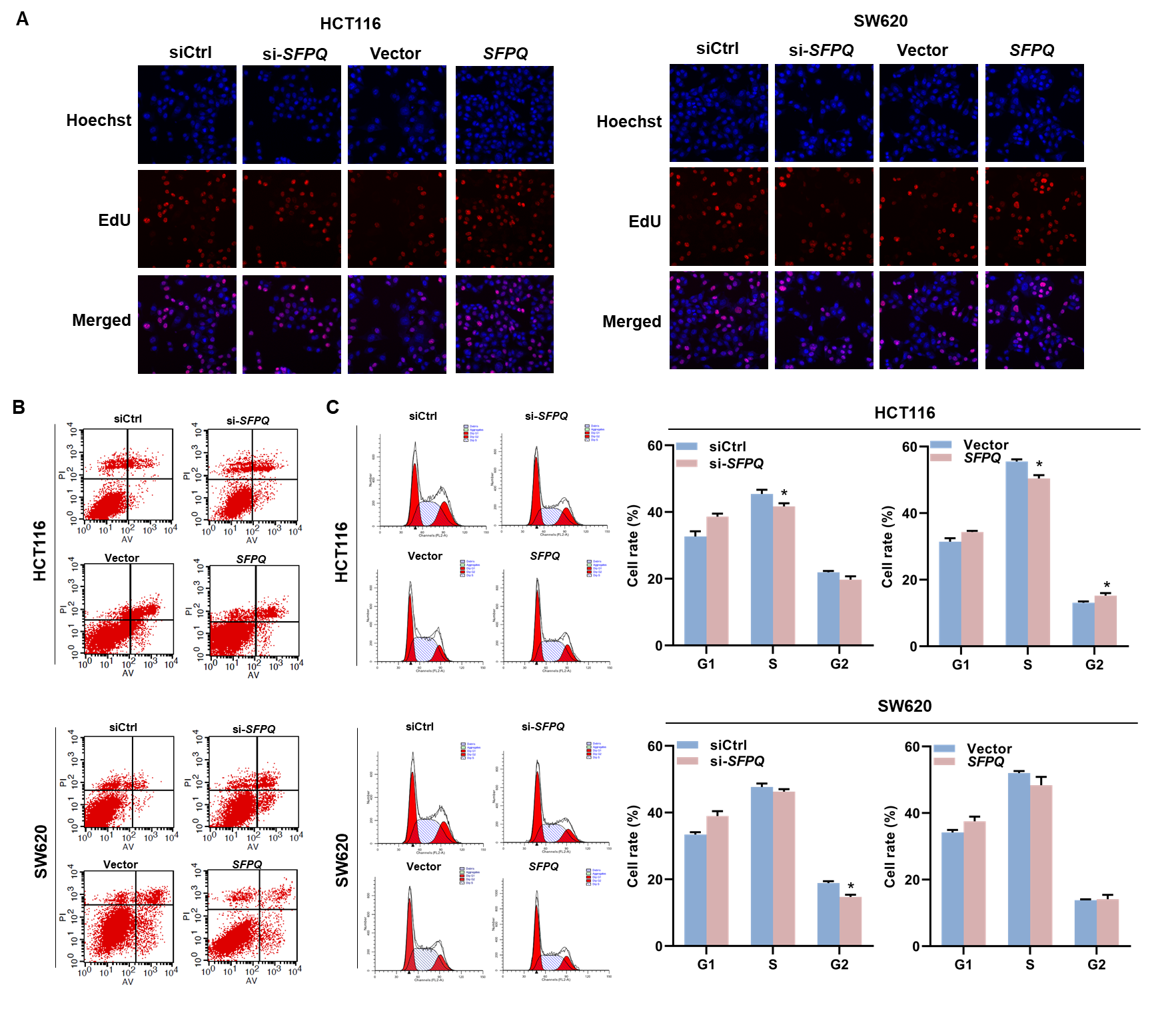
**

**Figure S4**

**
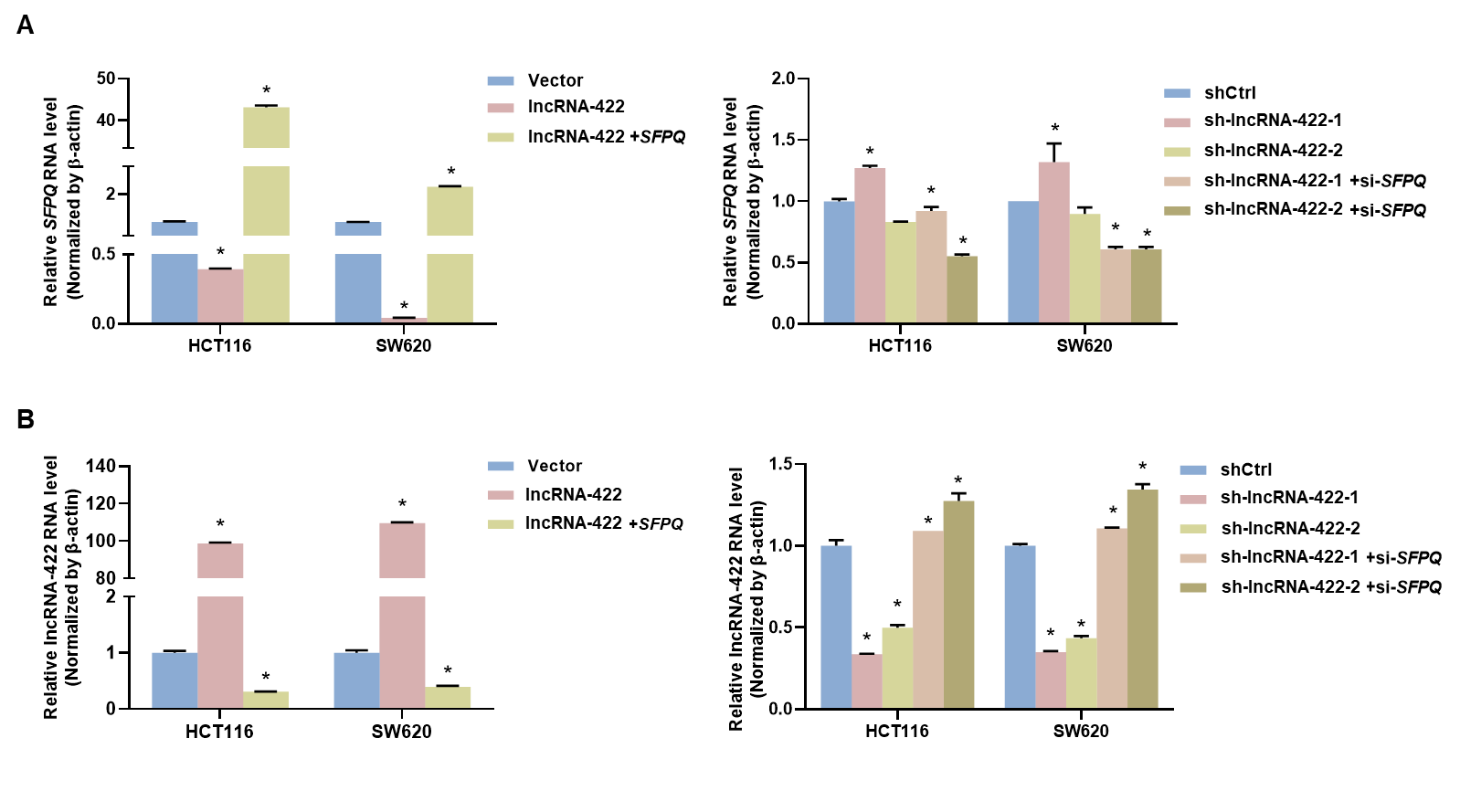
**

**Figure S5**

**
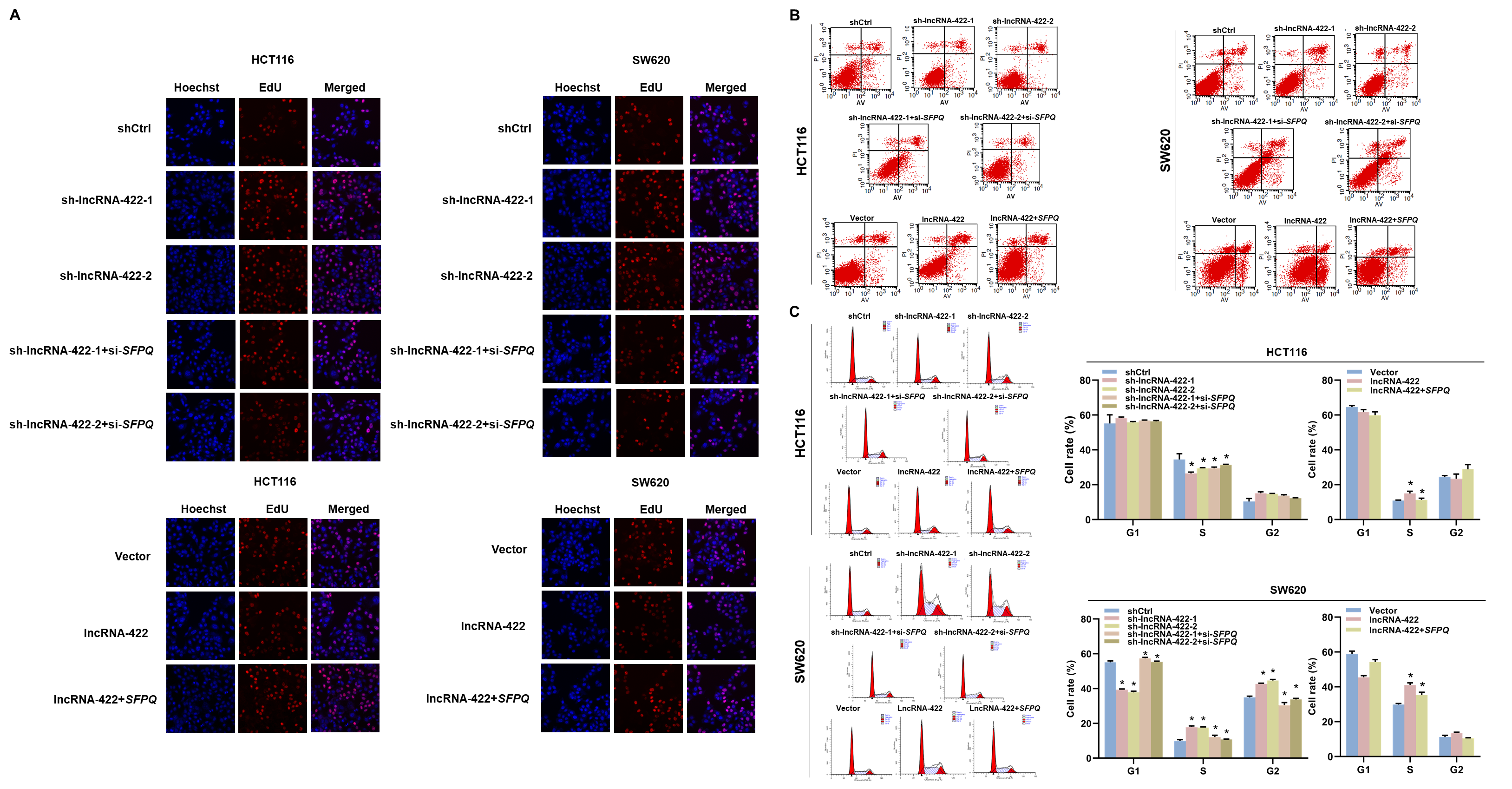
**

**Figure S6**

**
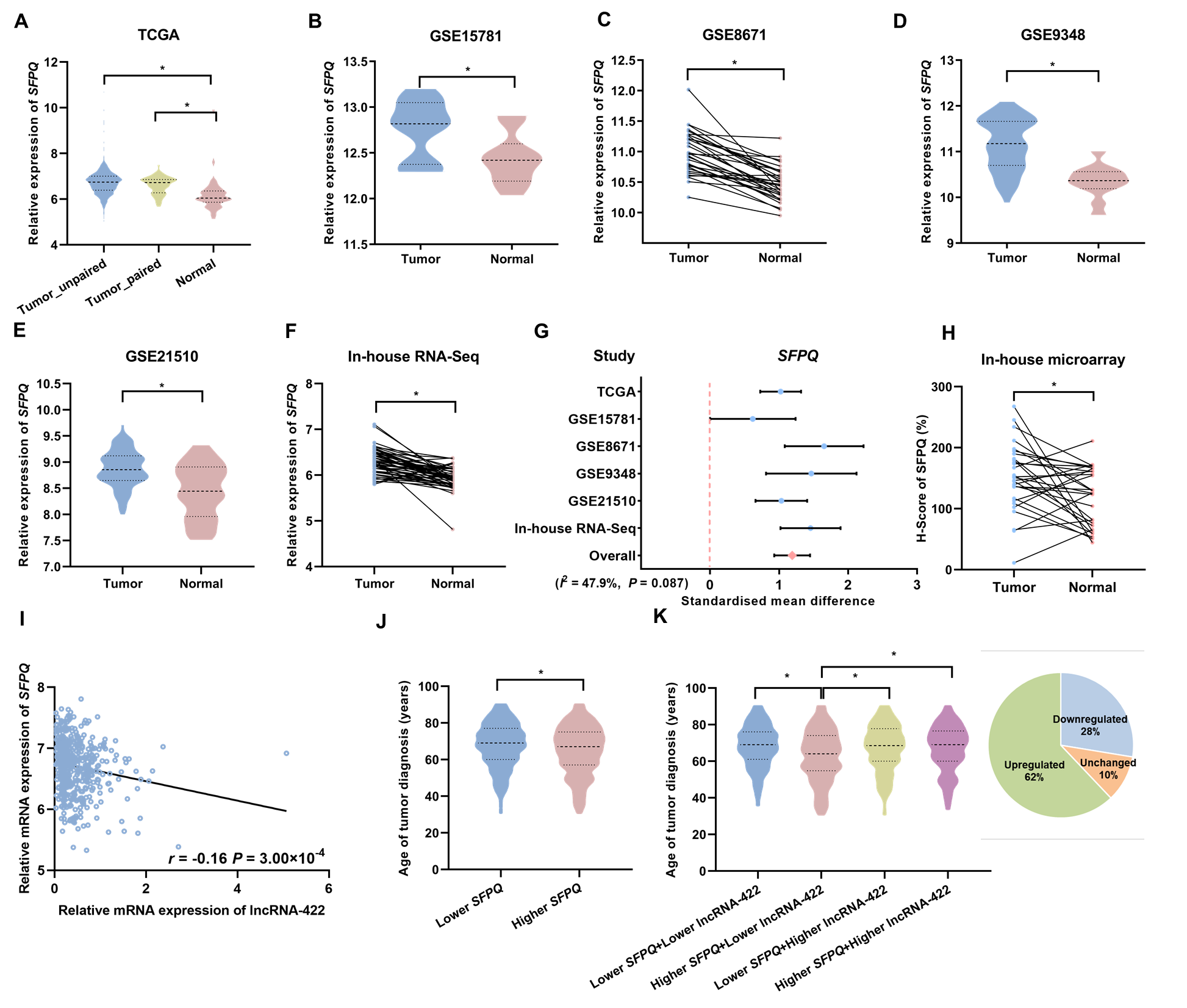
**

**Figure S7**


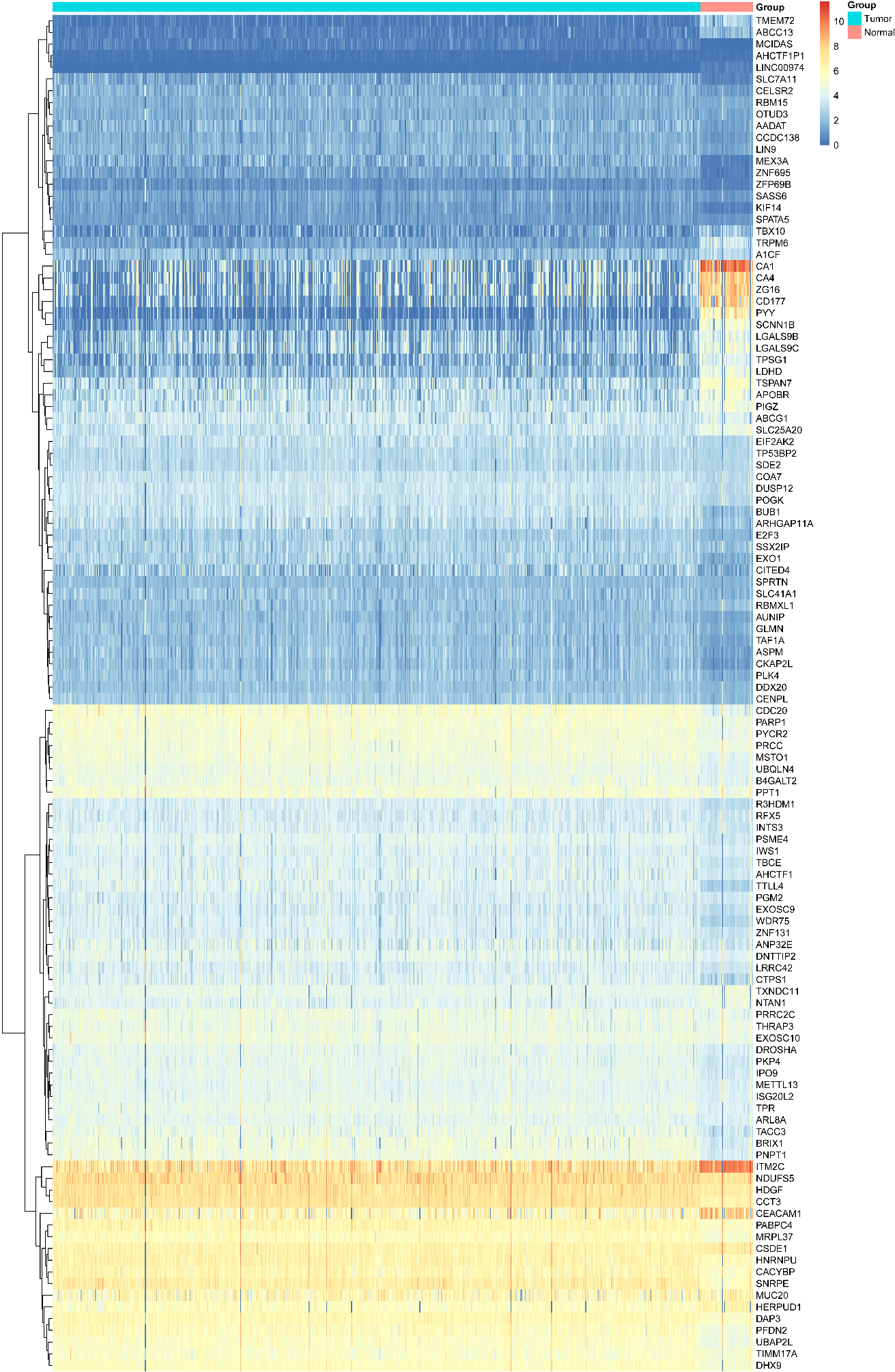


**Figure S8**

**
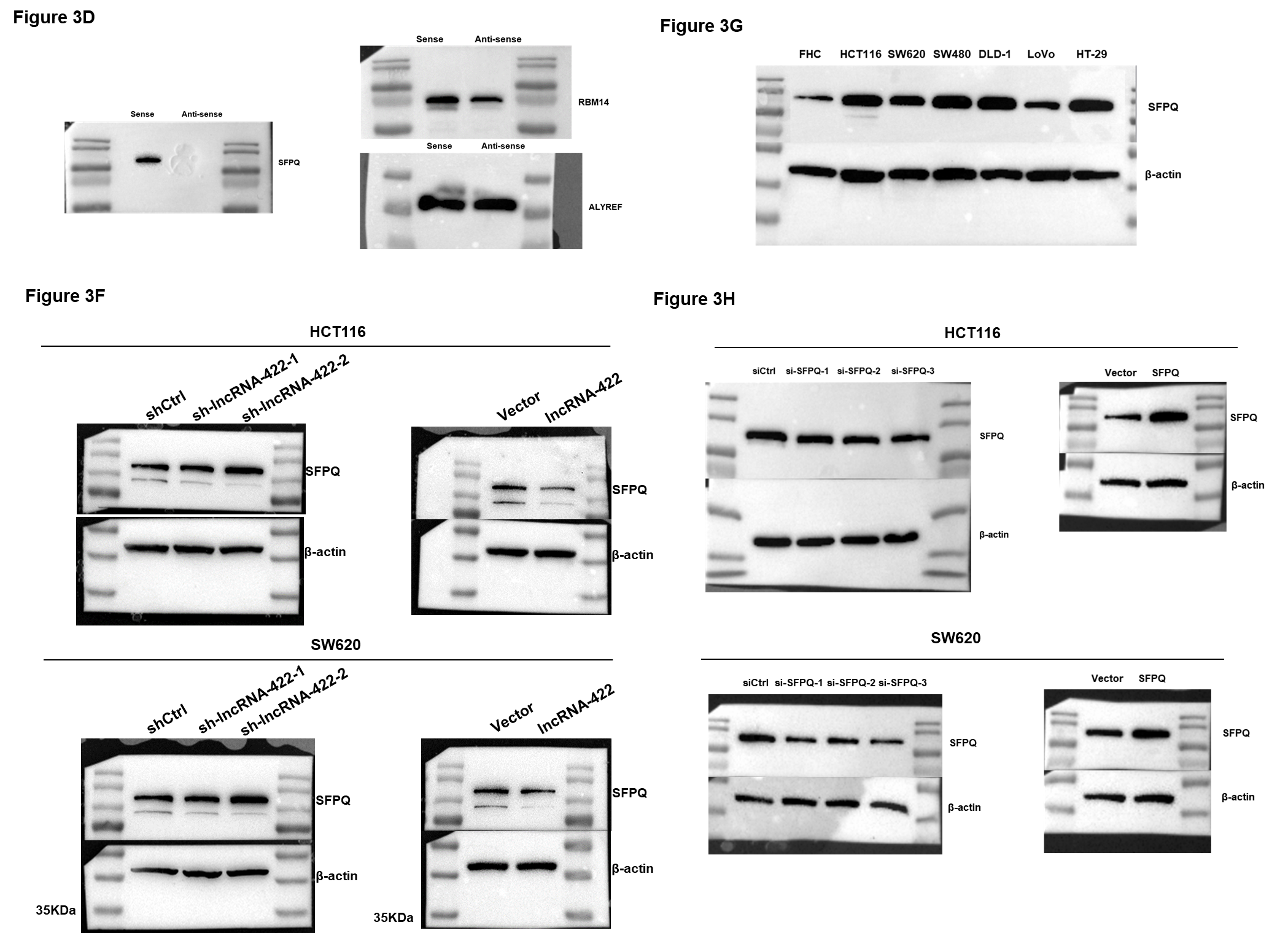
**

**Table S1.** Relationships between lncRNA-422 or *SFPQ* expression and clinicopathologic factors of the patients with colorectal cancer

| Characteristics | lncRNA-422 | | *P* value | *SFPQ* | | *P* value |
| --- | --- | --- | --- | --- | --- | --- |
|  | Low | High |  | High | Low |  |
| Ages (years) |  |  | **0.040** |  |  | **<0.001** |
| <60 | 101 | 77 |  | 106 | 72 |  |
| ≥60 | 218 | 245 |  | 214 | 249 | |
| Gender |  |  | 0.460 |  |  | 0.720 |
| Male | 165 | 177 |  | 168 | 174 | |
| Female | 154 | 145 |  | 152 | 147 | |
| Family history |  |  | 1.000 |  |  | 0.940 |
| Yes | 39 | 38 |  | 36 | 41 | |
| No | 242 | 230 |  | 215 | 257 |  |
| Tumor site |  |  | 0.850 |  |  | 1.000 |
| Colon | 237 | 234 |  | 236 | 235 | |
| Rectum | 78 | 81 |  | 80 | 79 |  |
| Tumor stage |  |  | 1.000 |  |  | 0.630 |
| I+II | 174 | 174 |  | 175 | 173 | |
| III+IV | 137 | 136 |  | 131 | 142 | |
| Depth of tumor | | | 0.560 |  |  | 0.900 |
| T1 | 9 | 11 |  | 10 | 10 |  |
| T2 | 52 | 59 |  | 53 | 58 |  |
| T3 | 214 | 220 |  | 221 | 213 | |
| T4 | 42 | 32 |  | 35 | 39 |  |
| Lymph node metastasis | |  | 0.670 |  | | 0.140 |
| Yes | 304 | 302 |  | 294 | 312 | |
| No | 11 | 8 |  | 13 | 6 |  |

**Table S2.** Mass Spectrometry protein identification results for biotinylated lncRNA-422 RNA pull-down experiments

| Gene names | Peptides | |  | Sequence coverage | |  | LFQ intensity | |  | Score (Peptides) | Score (Sequence coverage) | Weight (kDa) | TCGA | |  | In-house RNA-Seq | |
| --- | --- | --- | --- | --- | --- | --- | --- | --- | --- | --- | --- | --- | --- | --- | --- | --- | --- |
|  | Sense | Antisense |  | Sense | Antisense |  | Sense | Antisense |  |  |  |  | FC | *P* |  | FC | *P* |
| ALYREF | 2 | 0 |  | 12.9 | 0 |  | 1965400 | 0 |  | 35.7 | 12.1 | 27.6 | 1.21 | 8.20E-14 |  | 1.22 | 4.15E-12 |
| ANKRD66 | 1 | 0 |  | 2.8 | 0 |  | 94884000 | 0 |  | 26.1 | 5.7 | 27.9 | 1.15 | 8.10E-01 |  | 0.41 | 4.84E-01 |
| C2CD3 | 1 | 0 |  | 2.2 | 0 |  | 95140000 | 0 |  | 24.2 | 6.7 | 75.6 | 1.17 | 1.29E-11 |  | 1.00 | 7.83E-01 |
| DDX17 | 1 | 0 |  | 2.6 | 0 |  | 14334000 | 0 |  | 92.6 | 18.9 | 47.6 | 1.04 | 7.34E-02 |  | 0.98 | 2.75E-02 |
| DISC1 | 1 | 0 |  | 2.4 | 0 |  | 29466000 | 0 |  | 26.8 | 5.7 | 67.1 | 0.99 | 9.06E-01 |  | 0.88 | 9.82E-02 |
| DRC7 | 1 | 0 |  | 1.9 | 0 |  | 17142000 | 0 |  | 34.9 | 5.7 | 50.9 | 1.6 | 2.56E-01 |  | 5.29 | 3.04E-01 |
| EDF1 | 2 | 0 |  | 3.3 | 0 |  | 5970600 | 0 |  | 24.9 | 12.5 | 16.4 | 0.99 | 9.81E-01 |  | 1.01 | 3.15E-01 |
| EXOSC7 | 1 | 0 |  | 7.3 | 0 |  | 5628500 | 0 |  | 36.3 | 6.5 | 17.9 | 1.28 | 4.14E-37 |  | 1.27 | 1.70E-10 |
| H1FX | 1 | 0 |  | 8.5 | 0 |  | 2562800 | 0 |  | 32.5 | 6.8 | 22.5 | 1.04 | 3.87E-02 |  | 1.00 | 7.86E-01 |
| HIST1H1E | 1 | 0 |  | 4.1 | 0 |  | 4374000 | 0 |  | 54.6 | 6.5 | 21.9 | 4.12 | 7.62E-03 |  | 1.06 | 4.87E-05 |
| JUP | 2 | 0 |  | 3.1 | 0 |  | 8797000 | 0 |  | 51.5 | 14.4 | 81.7 | 1.07 | 2.97E-03 |  | 1.03 | 7.25E-02 |
| PYGO2 | 1 | 0 |  | 7.1 | 0 |  | 11980000 | 0 |  | 15.8 | 5.9 | 41.2 | 1.01 | 4.61E-01 |  | 1.04 | 2.90E-02 |
| RBM14 | 2 | 0 |  | 14.9 | 0 |  | 9246200 | 0 |  | 66.6 | 14.4 | 69.5 | 1.14 | 9.42E-10 |  | 1.07 | 7.66E-03 |
| RBM4 | 1 | 0 |  | 3.8 | 0 |  | 667960 | 0 |  | 29.1 | 6.1 | 26.3 | 1.07 | 1.67E-04 |  | 1.01 | 3.59E-01 |
| RPA1 | 1 | 0 |  | 2.6 | 0 |  | 236000000 | 0 |  | 100.1 | 29.8 | 68.1 | 1.02 | 2.47E-01 |  | 1.03 | 9.65E-02 |
| RPA2 | 1 | 0 |  | 14 | 0 |  | 23807000 | 0 |  | 27.9 | 7.7 | 28.5 | 1.06 | 1.21E-03 |  | 1.1 | 3.07E-07 |
| RPS24 | 1 | 0 |  | 6.1 | 0 |  | 1619200 | 0 |  | 27.3 | 5.9 | 15.2 | 1.06 | 2.04E-05 |  | 1.07 | 1.93E-10 |
| RPS7 | 1 | 0 |  | 3.7 | 0 |  | 6688100 | 0 |  | 68.9 | 6.8 | 21.3 | 1.08 | 1.25E-10 |  | 1.09 | 8.52E-12 |
| SF3A2 | 1 | 0 |  | 7.4 | 0 |  | 3413700 | 0 |  | 14.7 | 5.6 | 15.2 | 1.16 | 2.44E-11 |  | 1.15 | 7.79E-09 |
| SFPQ | 2 | 0 |  | 10.8 | 0 |  | 14358000 | 0 |  | 29.9 | 12.3 | 95.4 | 1.08 | 2.56E-11 |  | 1.06 | 2.95E-10 |
| SSRP1 | 1 | 0 |  | 2.0 | 0 |  | 14646000 | 0 |  | 67.7 | 11.4 | 81.1 | 1.15 | 1.92E-11 |  | 1.14 | 8.31E-13 |
| SWT1 | 1 | 0 |  | 7.5 | 0 |  | 4983900 | 0 |  | 19.3 | 5.7 | 17.7 | 0.85 | 1.46E-03 |  | 0.82 | 9.27E-08 |
| TMEM101 | 1 | 0 |  | 4.1 | 0 |  | 593330 | 0 |  | 31.3 | 5.9 | 19.2 | 1.11 | 5.32E-05 |  | 1.02 | 3.73E-01 |

MW, Molecular Weight; FC, Fold Change.

**Table S3.** Co-expression analyses reveal 116 genes directly correlated by lncRNA-422 and *SFPQ*

| Symbol | TCGA | | | | |  | In-house RNA-Seq | | | | |
| --- | --- | --- | --- | --- | --- | --- | --- | --- | --- | --- | --- |
|  | lncRNA-422 | |  | *SFPQ* | |  | lncRNA-422 | |  | *SFPQ* | |
|  | *r* | *P* |  | *r* | *P* |  | *r* | *P* |  | *r* | *P* |
| *A1CF* | 0.22 | 8.44E-09 |  | -0.15 | 5.27E-05 |  | 0.35 | 1.21E-02 |  | -0.28 | 4.16E-02 |
| *AADAT* | -0.16 | 1.89E-05 |  | 0.12 | 1.38E-03 |  | -0.41 | 2.39E-03 |  | 0.38 | 5.02E-03 |
| *ABCC13* | 0.31 | 5.08E-17 |  | -0.12 | 2.12E-03 |  | 0.32 | 2.09E-02 |  | -0.30 | 2.91E-02 |
| *ABCG1* | 0.15 | 5.29E-05 |  | -0.23 | 1.63E-09 |  | 0.29 | 3.48E-02 |  | -0.37 | 6.44E-03 |
| *AHCTF1* | -0.09 | 1.68E-02 |  | 0.44 | 6.83E-34 |  | -0.42 | 2.20E-03 |  | 0.47 | 4.87E-04 |
| *AHCTF1P1* | -0.08 | 4.45E-02 |  | 0.08 | 3.11E-02 |  | -0.40 | 3.61E-03 |  | 0.46 | 6.31E-04 |
| *ANP32E* | -0.14 | 2.16E-04 |  | 0.52 | 7.29E-49 |  | -0.30 | 3.00E-02 |  | 0.57 | 9.81E-06 |
| *APOBR* | 0.28 | 1.30E-13 |  | -0.11 | 3.21E-03 |  | 0.29 | 3.67E-02 |  | -0.27 | 4.90E-02 |
| *ARHGAP11A* | -0.12 | 2.31E-03 |  | 0.09 | 1.56E-02 |  | -0.36 | 7.81E-03 |  | 0.48 | 3.20E-04 |
| *ARL8A* | -0.09 | 2.30E-02 |  | 0.42 | 1.94E-31 |  | -0.31 | 2.33E-02 |  | 0.29 | 3.45E-02 |
| *ASPM* | -0.08 | 2.81E-02 |  | 0.37 | 1.58E-24 |  | -0.29 | 3.89E-02 |  | 0.54 | 3.50E-05 |
| *AUNIP* | -0.10 | 1.03E-02 |  | 0.86 | 4.94E-203 |  | -0.36 | 8.79E-03 |  | 0.45 | 7.35E-04 |
| *B4GALT2* | -0.14 | 2.55E-04 |  | 0.80 | 9.15E-157 |  | -0.32 | 2.23E-02 |  | 0.38 | 4.99E-03 |
| *BRIX1* | -0.13 | 8.82E-04 |  | 0.14 | 1.54E-04 |  | -0.30 | 2.87E-02 |  | 0.52 | 6.65E-05 |
| *BUB1* | -0.14 | 1.81E-04 |  | 0.32 | 1.43E-17 |  | -0.30 | 3.25E-02 |  | 0.52 | 7.67E-05 |
| *CA1* | 0.27 | 4.05E-13 |  | -0.12 | 1.33E-03 |  | 0.30 | 2.80E-02 |  | -0.28 | 4.49E-02 |
| *CA4* | 0.30 | 6.71E-16 |  | -0.15 | 1.05E-04 |  | 0.29 | 3.74E-02 |  | -0.37 | 7.55E-03 |
| *CACYBP* | -0.10 | 9.53E-03 |  | 0.49 | 4.77E-44 |  | -0.34 | 1.46E-02 |  | 0.37 | 7.03E-03 |
| *CCDC138* | -0.15 | 6.81E-05 |  | 0.27 | 8.39E-13 |  | -0.41 | 2.69E-03 |  | 0.54 | 3.12E-05 |
| *CCT3* | -0.16 | 2.25E-05 |  | 0.55 | 2.61E-55 |  | -0.35 | 9.91E-03 |  | 0.48 | 2.90E-04 |
| *CD177* | 0.24 | 8.27E-11 |  | -0.12 | 2.06E-03 |  | 0.31 | 2.74E-02 |  | -0.28 | 4.59E-02 |
| *CDC20* | -0.12 | 1.97E-03 |  | 0.61 | 1.55E-73 |  | -0.31 | 2.78E-02 |  | 0.46 | 5.26E-04 |
| *CEACAM1* | 0.24 | 2.33E-10 |  | -0.12 | 1.63E-03 |  | 0.29 | 3.43E-02 |  | -0.28 | 4.33E-02 |
| *CELSR2* | -0.14 | 1.43E-04 |  | 0.63 | 2.63E-79 |  | -0.28 | 4.37E-02 |  | 0.32 | 1.97E-02 |
| *CENPL* | -0.09 | 1.53E-02 |  | 0.44 | 1.95E-34 |  | -0.30 | 3.31E-02 |  | 0.37 | 6.74E-03 |
| *CITED4* | -0.19 | 3.14E-07 |  | 0.13 | 6.63E-04 |  | -0.34 | 1.24E-02 |  | 0.31 | 2.56E-02 |
| *CKAP2L* | -0.09 | 1.69E-02 |  | 0.31 | 2.48E-17 |  | -0.28 | 4.75E-02 |  | 0.43 | 1.60E-03 |
| *COA7* | -0.12 | 2.25E-03 |  | 0.94 | 0.00E+00 |  | -0.29 | 3.95E-02 |  | 0.52 | 8.58E-05 |
| *CSDE1* | -0.10 | 9.10E-03 |  | 0.58 | 4.39E-64 |  | -0.29 | 3.75E-02 |  | 0.31 | 2.72E-02 |
| *CTPS1* | -0.16 | 2.61E-05 |  | 0.86 | 4.25E-208 |  | -0.35 | 1.05E-02 |  | 0.70 | 8.37E-09 |
| *DAP3* | -0.13 | 5.80E-04 |  | 0.56 | 8.92E-58 |  | -0.37 | 7.41E-03 |  | 0.53 | 5.48E-05 |
| *DDX20* | -0.15 | 6.28E-05 |  | 0.58 | 9.21E-64 |  | -0.28 | 4.34E-02 |  | 0.66 | 9.88E-08 |
| *DHX9* | -0.18 | 1.86E-06 |  | 0.47 | 2.30E-40 |  | -0.32 | 1.93E-02 |  | 0.60 | 2.72E-06 |
| *DNTTIP2* | -0.08 | 4.66E-02 |  | 0.90 | 7.15E-249 |  | -0.36 | 7.83E-03 |  | 0.59 | 3.45E-06 |
| *DROSHA* | -0.15 | 4.39E-05 |  | 0.16 | 2.22E-05 |  | -0.38 | 4.93E-03 |  | 0.32 | 2.16E-02 |
| *DUSP12* | -0.14 | 2.41E-04 |  | 0.55 | 2.15E-56 |  | -0.39 | 4.37E-03 |  | 0.41 | 2.75E-03 |
| *E2F3* | -0.15 | 7.34E-05 |  | 0.14 | 1.59E-04 |  | -0.29 | 3.59E-02 |  | 0.45 | 8.92E-04 |
| *EIF2AK2* | -0.11 | 5.48E-03 |  | 0.38 | 6.29E-25 |  | -0.33 | 1.59E-02 |  | 0.29 | 3.48E-02 |
| *EXO1* | -0.16 | 3.12E-05 |  | 0.37 | 4.30E-24 |  | -0.29 | 3.70E-02 |  | 0.57 | 9.30E-06 |
| *EXOSC10* | -0.07 | 4.86E-02 |  | 0.82 | 8.10E-172 |  | -0.31 | 2.62E-02 |  | 0.30 | 3.15E-02 |
| *EXOSC9* | -0.08 | 3.19E-02 |  | 0.16 | 1.30E-05 |  | -0.39 | 4.09E-03 |  | 0.37 | 6.20E-03 |
| *GLMN* | -0.08 | 3.68E-02 |  | 0.83 | 1.52E-179 |  | -0.35 | 1.07E-02 |  | 0.52 | 7.24E-05 |
| *HDGF* | -0.13 | 3.62E-04 |  | 0.59 | 5.60E-67 |  | -0.42 | 2.08E-03 |  | 0.34 | 1.27E-02 |
| *HERPUD1* | 0.20 | 6.23E-08 |  | -0.27 | 2.25E-13 |  | 0.29 | 3.77E-02 |  | -0.40 | 2.98E-03 |
| *HNRNPU* | -0.15 | 7.91E-05 |  | 0.51 | 5.63E-48 |  | -0.30 | 3.18E-02 |  | 0.58 | 7.83E-06 |
| *INTS3* | -0.13 | 8.68E-04 |  | 0.57 | 4.52E-62 |  | -0.30 | 3.35E-02 |  | 0.28 | 4.82E-02 |
| *IPO9* | -0.15 | 4.85E-05 |  | 0.47 | 5.81E-40 |  | -0.40 | 3.07E-03 |  | 0.38 | 5.29E-03 |
| *ISG20L2* | -0.15 | 4.23E-05 |  | 0.56 | 2.92E-59 |  | -0.38 | 5.08E-03 |  | 0.36 | 9.63E-03 |
| *ITM2C* | 0.21 | 1.67E-08 |  | -0.12 | 2.01E-03 |  | 0.28 | 4.79E-02 |  | -0.31 | 2.33E-02 |
| *IWS1* | -0.12 | 2.23E-03 |  | 0.33 | 2.25E-19 |  | -0.33 | 1.87E-02 |  | 0.47 | 5.03E-04 |
| *KIF14* | -0.11 | 5.18E-03 |  | 0.33 | 2.90E-19 |  | -0.35 | 1.18E-02 |  | 0.51 | 9.72E-05 |
| *LDHD* | 0.31 | 5.89E-17 |  | -0.18 | 2.71E-06 |  | 0.44 | 1.17E-03 |  | -0.28 | 4.60E-02 |
| *LGALS9B* | 0.12 | 2.13E-03 |  | -0.13 | 5.75E-04 |  | 0.30 | 3.19E-02 |  | -0.37 | 6.87E-03 |
| *LGALS9C* | 0.13 | 6.90E-04 |  | -0.12 | 1.94E-03 |  | 0.36 | 9.34E-03 |  | -0.38 | 5.19E-03 |
| *LIN9* | -0.09 | 2.35E-02 |  | 0.35 | 4.70E-21 |  | -0.37 | 6.41E-03 |  | 0.53 | 5.47E-05 |
| *LINC00974* | 0.30 | 1.96E-16 |  | -0.13 | 3.92E-04 |  | 0.31 | 2.63E-02 |  | -0.32 | 2.04E-02 |
| *LRRC42* | -0.08 | 2.73E-02 |  | 0.87 | 9.04E-216 |  | -0.27 | 4.85E-02 |  | 0.38 | 5.80E-03 |
| *MCIDAS* | -0.14 | 1.81E-04 |  | 0.12 | 1.58E-03 |  | -0.37 | 7.49E-03 |  | 0.41 | 2.26E-03 |
| *METTL13* | -0.16 | 2.46E-05 |  | 0.56 | 2.25E-58 |  | -0.29 | 3.69E-02 |  | 0.33 | 1.69E-02 |
| *MEX3A* | -0.24 | 2.69E-10 |  | 0.20 | 5.78E-08 |  | -0.30 | 3.10E-02 |  | 0.38 | 5.11E-03 |
| *MRPL37* | -0.13 | 8.54E-04 |  | 0.82 | 1.48E-173 |  | -0.32 | 2.13E-02 |  | 0.47 | 3.83E-04 |
| *MSTO1* | -0.17 | 6.14E-06 |  | 0.55 | 6.61E-56 |  | -0.36 | 8.28E-03 |  | 0.36 | 9.70E-03 |
| *MUC20* | 0.13 | 5.30E-04 |  | -0.08 | 3.70E-02 |  | 0.30 | 2.95E-02 |  | -0.40 | 3.15E-03 |
| *NDUFS5* | -0.08 | 3.31E-02 |  | 0.77 | 7.25E-140 |  | -0.33 | 1.85E-02 |  | 0.61 | 1.55E-06 |
| *NTAN1* | 0.08 | 2.70E-02 |  | -0.22 | 2.65E-09 |  | 0.29 | 3.80E-02 |  | -0.28 | 4.33E-02 |
| *OTUD3* | -0.09 | 1.31E-02 |  | 0.79 | 1.42E-148 |  | -0.29 | 3.57E-02 |  | 0.37 | 7.30E-03 |
| *PABPC4* | -0.13 | 8.67E-04 |  | 0.86 | 1.22E-209 |  | -0.32 | 2.18E-02 |  | 0.53 | 4.42E-05 |
| *PARP1* | -0.18 | 1.54E-06 |  | 0.45 | 3.05E-36 |  | -0.39 | 4.44E-03 |  | 0.36 | 8.09E-03 |
| *PFDN2* | -0.12 | 2.18E-03 |  | 0.48 | 2.13E-42 |  | -0.41 | 2.60E-03 |  | 0.35 | 1.17E-02 |
| *PGM2* | -0.12 | 2.18E-03 |  | 0.12 | 2.32E-03 |  | -0.37 | 7.12E-03 |  | 0.32 | 2.13E-02 |
| *PIGZ* | 0.18 | 2.57E-06 |  | -0.10 | 6.83E-03 |  | 0.30 | 3.04E-02 |  | -0.28 | 4.54E-02 |
| *PKP4* | -0.13 | 5.79E-04 |  | 0.30 | 5.18E-16 |  | -0.29 | 3.94E-02 |  | 0.49 | 2.01E-04 |
| *PLK4* | -0.13 | 8.59E-04 |  | 0.19 | 3.77E-07 |  | -0.31 | 2.74E-02 |  | 0.44 | 1.17E-03 |
| *PNPT1* | -0.17 | 8.92E-06 |  | 0.17 | 1.17E-05 |  | -0.27 | 4.85E-02 |  | 0.34 | 1.29E-02 |
| *POGK* | -0.15 | 1.08E-04 |  | 0.58 | 8.23E-65 |  | -0.34 | 1.45E-02 |  | 0.29 | 3.42E-02 |
| *PPT1* | -0.08 | 2.79E-02 |  | 0.84 | 2.14E-187 |  | -0.49 | 2.35E-04 |  | 0.27 | 4.96E-02 |
| *PRCC* | -0.10 | 7.95E-03 |  | 0.57 | 6.16E-61 |  | -0.34 | 1.34E-02 |  | 0.27 | 4.99E-02 |
| *PRRC2C* | -0.13 | 7.31E-04 |  | 0.55 | 1.65E-56 |  | -0.41 | 2.43E-03 |  | 0.42 | 2.01E-03 |
| *PSME4* | -0.11 | 5.13E-03 |  | 0.29 | 8.92E-15 |  | -0.29 | 3.67E-02 |  | 0.30 | 2.99E-02 |
| *PYCR2* | -0.12 | 1.21E-03 |  | 0.40 | 1.28E-28 |  | -0.34 | 1.33E-02 |  | 0.40 | 3.61E-03 |
| *PYY* | 0.19 | 2.64E-07 |  | -0.10 | 8.14E-03 |  | 0.30 | 2.84E-02 |  | -0.28 | 4.12E-02 |
| *R3HDM1* | -0.19 | 3.71E-07 |  | 0.16 | 2.71E-05 |  | -0.28 | 4.15E-02 |  | 0.39 | 4.29E-03 |
| *RBM15* | -0.11 | 5.29E-03 |  | 0.50 | 6.73E-46 |  | -0.41 | 2.58E-03 |  | 0.31 | 2.79E-02 |
| *RBMXL1* | -0.11 | 3.18E-03 |  | 0.91 | 1.91E-261 |  | -0.32 | 1.88E-02 |  | 0.36 | 8.51E-03 |
| *RFX5* | -0.18 | 2.43E-06 |  | 0.53 | 1.02E-52 |  | -0.37 | 7.13E-03 |  | 0.28 | 4.57E-02 |
| *SASS6* | -0.11 | 5.44E-03 |  | 0.88 | 2.61E-225 |  | -0.30 | 3.14E-02 |  | 0.70 | 1.07E-08 |
| *SCNN1B* | 0.28 | 7.37E-14 |  | -0.13 | 6.65E-04 |  | 0.32 | 1.91E-02 |  | -0.31 | 2.46E-02 |
| *SDE2* | -0.09 | 1.46E-02 |  | 0.35 | 5.70E-22 |  | -0.37 | 7.00E-03 |  | 0.34 | 1.37E-02 |
| *SLC25A20* | 0.26 | 2.21E-12 |  | -0.12 | 1.68E-03 |  | 0.41 | 2.31E-03 |  | -0.30 | 2.94E-02 |
| *SLC41A1* | -0.20 | 6.63E-08 |  | 0.35 | 1.18E-21 |  | -0.49 | 2.26E-04 |  | 0.37 | 7.31E-03 |
| *SLC7A11* | -0.09 | 1.90E-02 |  | 0.09 | 1.73E-02 |  | -0.30 | 2.97E-02 |  | 0.32 | 2.12E-02 |
| *SNRPE* | -0.09 | 1.21E-02 |  | 0.31 | 1.40E-16 |  | -0.28 | 4.50E-02 |  | 0.43 | 1.39E-03 |
| *SPATA5* | -0.09 | 1.71E-02 |  | 0.10 | 6.89E-03 |  | -0.39 | 4.73E-03 |  | 0.36 | 8.88E-03 |
| *SPRTN* | -0.14 | 2.63E-04 |  | 0.37 | 1.11E-24 |  | -0.32 | 2.24E-02 |  | 0.29 | 3.53E-02 |
| *SSX2IP* | -0.13 | 5.33E-04 |  | 0.83 | 1.01E-177 |  | -0.42 | 2.19E-03 |  | 0.41 | 2.77E-03 |
| *TACC3* | -0.10 | 7.24E-03 |  | 0.22 | 2.41E-09 |  | -0.28 | 4.53E-02 |  | 0.41 | 2.46E-03 |
| *TAF1A* | -0.11 | 3.85E-03 |  | 0.39 | 1.47E-26 |  | -0.32 | 2.23E-02 |  | 0.50 | 1.76E-04 |
| *TBCE* | -0.10 | 1.00E-02 |  | 0.43 | 5.96E-33 |  | -0.31 | 2.78E-02 |  | 0.39 | 4.66E-03 |
| *TBX10* | 0.16 | 2.37E-05 |  | -0.13 | 5.83E-04 |  | 0.38 | 5.01E-03 |  | -0.33 | 1.79E-02 |
| *THRAP3* | -0.08 | 3.72E-02 |  | 0.88 | 1.02E-230 |  | -0.29 | 3.45E-02 |  | 0.73 | 1.15E-09 |
| *TIMM17A* | -0.10 | 6.17E-03 |  | 0.35 | 9.10E-22 |  | -0.34 | 1.23E-02 |  | 0.32 | 2.22E-02 |
| *TMEM72* | 0.24 | 1.34E-10 |  | -0.13 | 3.53E-04 |  | 0.28 | 4.19E-02 |  | -0.28 | 4.07E-02 |
| *TP53BP2* | -0.13 | 7.35E-04 |  | 0.45 | 3.47E-36 |  | -0.43 | 1.64E-03 |  | 0.35 | 1.10E-02 |
| *TPR* | -0.15 | 9.40E-05 |  | 0.44 | 1.00E-33 |  | -0.31 | 2.39E-02 |  | 0.54 | 3.47E-05 |
| *TPSG1* | 0.26 | 1.58E-12 |  | -0.13 | 8.67E-04 |  | 0.29 | 3.83E-02 |  | -0.35 | 1.17E-02 |
| *TRPM6* | 0.24 | 2.64E-10 |  | -0.10 | 1.03E-02 |  | 0.29 | 3.50E-02 |  | -0.30 | 3.24E-02 |
| *TSPAN7* | 0.28 | 2.50E-14 |  | -0.18 | 2.08E-06 |  | 0.33 | 1.54E-02 |  | -0.32 | 2.17E-02 |
| *TTLL4* | -0.19 | 3.64E-07 |  | 0.10 | 1.18E-02 |  | -0.34 | 1.52E-02 |  | 0.38 | 5.06E-03 |
| *TXNDC11* | 0.23 | 1.38E-09 |  | -0.29 | 4.87E-15 |  | 0.40 | 3.05E-03 |  | -0.32 | 2.17E-02 |
| *UBAP2L* | -0.21 | 2.84E-08 |  | 0.54 | 6.73E-55 |  | -0.35 | 1.09E-02 |  | 0.30 | 3.18E-02 |
| *UBQLN4* | -0.18 | 2.91E-06 |  | 0.50 | 2.56E-45 |  | -0.40 | 3.59E-03 |  | 0.45 | 7.10E-04 |
| *WDR75* | -0.14 | 1.23E-04 |  | 0.20 | 1.98E-07 |  | -0.31 | 2.33E-02 |  | 0.55 | 2.03E-05 |
| *ZFP69B* | -0.13 | 9.34E-04 |  | 0.82 | 1.97E-171 |  | -0.33 | 1.81E-02 |  | 0.74 | 2.54E-10 |
| *ZG16* | 0.27 | 1.47E-13 |  | -0.14 | 2.70E-04 |  | 0.33 | 1.58E-02 |  | -0.32 | 2.29E-02 |
| *ZNF131* | -0.12 | 2.06E-03 |  | 0.17 | 9.84E-06 |  | -0.31 | 2.50E-02 |  | 0.34 | 1.52E-02 |
| *ZNF695* | -0.12 | 1.30E-03 |  | 0.31 | 2.81E-17 |  | -0.36 | 8.60E-03 |  | 0.52 | 7.58E-05 |

**Table S4.** Sequences of primers and probes used in this study

| **Gene** | **Sequence (5’-3’)** |
| --- | --- |
| 5’ RACE | F: GTTCAGCCAGGGTTGACATCACTGCTT |
|  | R: GTCCTGAAGCCCAGGATGAGTAAGACAC |
| 3’ RACE | F: CCTGGGCTTCAGGACATCTGTGTAACAGA |
|  | R: GGCTGAACACTGGAATCACCTGGGAAGA |
| β-actin | F: CATGTACGTTGCTATCCAGGC |
|  | R: CTCCTTAATGTCACGCACGAT |
| lncRNA-422 | F: CCAAAGGGGAAGAGATGCGG |
|  | R: CCCTGCCCCGAAAATTGAAA |
| GAPDH | F: CATGAGAAGTATGACAACAGCCT |
|  | R: AGTCCTTCCACGATACCAAAGT |
| U6 | RT: AAAATATGGAACGCTTCACG |
|  | F: CGCTTCGGCAGCACATATACTAAAATTGGAAC |
|  | R: GCTTCACGAATTTGCGTGTCATCCTTGC |
| *SFPQ* | F: GACAGCTTCGAGTTCGCTTTG |
|  | R: TCATCCACTATTACAACAGCCCT |
| *MEX3A* | F: TGGAGAACTAGGATGTTTCGGG |
|  | R: GAGGCAGAGTTGATCGAGAGC |
| *SLC41A1* | F: AGGTCTTCATCCTAGTGCCTG |
|  | R: CCATGTGTCCAATGTTGGCTG |
| *UBAP2L* | F: AGCCGTGGACGAGAGTTTC |
|  | R: CGTATTGCCGCTGCTATTGC |
| lncRNA-422 | Sense: GUUUCCAGUUCUUUGCUCCGCAUCU |
|  | Antisense: AGAUGCGGAGCAAAGAACUGGAAAC |
| sh-lncRNA-422-1 | GGTGAAGCAGTGATGTCAA |
| sh-lncRNA-422-2 | GCAACACCTCCTGCATCTA |
| sh-lncRNA-422-3 | GCAAGGTTGAGAACTACTT |
| si-*SFPQ*-1 | GGAAGAAGCCTTTAGCCAA |
| si-*SFPQ*-2 | GGAAGATGCCTATCATGAA |
| si-*SFPQ*-3 | CCATGATGGGAAGTGACAT |

**Supplemental Materials and Methods**

**Cell culture**

HCT116 cells were obtained from American Type Culture Collection (ATCC) and grown in McCoy’s medium (Gibco, CA, USA) with 10% fetal serum (FBS) and 1% penicillin/streptomycin. Other colorectal cancer cells and colon epithelial cells including SW620, SW480, DLD-1, LoVo and FHC cells, were also used. All cell lines were cultured in an incubator at 37°C with high humidity and 5% CO_2_. All cells were routinely tested by DNA sequencing using the AmpF/STR method (Applied Biosystems, CA, USA) and confirmed to be mycoplasma-free (MycoAlert, MD, USA).

**Data collection**

Both colon adenocarcinoma (COAD) and rectum adenocarcinoma (READ) datasets, including mRNA and lncRNA data, were obtained from The Cancer Genome Atlas (TCGA) and Gene Expression Omnibus (GEO) databases under the accession numbers GSE104836, GSE15781, GSE8671, GSE9348, and GSE21510. Colon of sigmoid and transverse samples were obtained from the Genotype-Tissue Expression (GTEx).

Independent cohorts including 52 colorectal cancer patients were recruited to generate data. A total of 52 paired colorectal cancer tissues and adjacent normal tissues were collected from patients during surgery at Affiliated Nanjing First Hospital of Nanjing Medical University (Nanjing, China). This study was approved by the ethics committee on Human Research of the Nanjing Medical University and written informed consent was gathered from all participants.

**Co-expression analyses**

Two separate correlation matrices were constructed, i.e., for lncRNA-422 and *SFPQ*. The Pearson correlation coefficient was used to calculate the correlations in TCGA and in-house RNA-Seq colorectal cancer tissues. A total of 89 genes with a significantly positive correlation *P* < 0.05 with *SFPQ* and significantly negative correlation *P* < 0.05 with lncRNA-422 were selected in both TCGA and in-house RNA-Seq, while 27 genes with a significantly negative correlation *P* < 0.05 with *SFPQ* and significantly positive correlation *P* < 0.05 with lncRNA-422 were selected in both databases. In general, 116 genes co-expressed with lncRNA-422 and *SFPQ* were selected for KEGG functional enrichment analysis.

**Prediction of the protein-coding potency of lncRNA-422**

LncRNA-422 was visualized on the UCSC browser ([http://genome.ucsc.edu](http://genome.ucsc.edu/)). We carried out a codon substitution frequency analysis by applying the software PhyloCSF to evaluate the coding capacity of lncRNA-422 in the transcript, based on evolutionary signatures in the 29-mammalian-genome alignment ^1^. In addition, the Coding Potential Alignment Tool (CPAT) ^2^, Coding Potential Calculator (CPC) ^3^, and Open Reading Frame (ORF) Finder (https://www.ncbi.nlm.nih.gov/orffinder/) were employed to estimate the protein-coding potentiality of lncRNA-422. UCA1 and HOTAIR acted as noncoding RNA controls, while ACTB and GAPDH acted as coding RNA controls.

**5′/3′ rapid ampliﬁcation of cDNA ends (RACE)**

Total RNA from colorectal cancer cells was used for first-strand cDNA synthesis. The SMARTer RACE cDNA Amplification kit (Clontech, California, USA) was able to ascertain 5′ and 3′ RACE by measuring the transcriptional initiation and termination sites of lncRNA-422 according to the manufacturer’s instructions. Briefly, clones per fragment were sequenced using standard Sanger sequencing. The gene-specific primers applied for 5′ and 3′ RACE are shown in **Table S4**.

**Plasmid construction and cell transfection**

The full-length lncRNA-422 sequence, the empty vector (Vector), the small hairpin RNA (shRNA) of lncRNA-422 (sh-lncRNA-422), and their negative control (shCtrl) were purchased from GeneChem (Shanghai, China). sh-lncRNA-422 was cloned into GV502 vectors (GeneChem, Shanghai, China) following cotransfection with the packaging plasmids (GeneChem) into 293T cells to construct lentiviral particles. In all, 72 h post-infection, stably transduced cells were selected with 2 μg/ml puromycin for 3 days. Next, lentiviruses were harvested after evaluating transfection efficiency by qPCR after 3 days. In addition, the pcDNA3.1-SFPQ plasmid (*SFPQ*) and empty plasmid (Vector) were constructed by Keygen Biotech (Nanjing, China). The small interfering RNA (siRNA) of *SFPQ* (si-*SFPQ*) and its negative control (siCtrl) were constructed by RiboBio (Shanghai, China).

**Quantitative mRNA analysis**

RNA was obtained using TRIzol reagent (Beyotime Biotechnology, Shanghai, China), and first-strand cDNA was generated using the High-Capacity cDNA Reverse Transcription Kit (Thermo Fisher Scientific, California, USA). For real-time PCR of lncRNA-422, primers were synthesized using SYBR qPCR Master Mix (Vazyme, Nanjing, China) in a LightCycler 480 II Real-Time PCR System (Roche, Rotkreuz, Switzerland). All the primer sequences used are listed in **Table S4**, and ACTB, GAPDH and U6 were used as internal controls, as appropriate.

**RNA fluorescence in situ hybridization (FISH)**

Cy3-labelled lncRNA lncRNA-422 probes were designed and synthesized by RiboBio (Shanghai, China). Subsequently, the signals of the probe were conducted using a Ribo fluorescence in situ hybridization kit (Shanghai, China) following the manufacturer’s directions and as previously described. All images were obtained via a confocal laser-scanning microscope (Nikon, Tokyo, Japan).

**Subcellular fractionation**

Cytoplasmic and nuclear fractions of the colorectal cancer cells were centrifuged, incubated, and extracted in the Nuclear/Cytoplasmic Isolation kit (Thermo Fisher Scientific, California, USA). Nuclear and cytoplasmic RNA was purified in the Nuclear and Cytoplasmic RNA Purification Kit (Invitrogen, CA, USA), and then GAPDH and U6 small nuclear RNA were used as the cytoplasmic and nuclear endogenous controls, respectively.

**RNA pull-down assay**

HCT116 cell lines expressing, the sense strand of lncRNA-422, and the antisense strand of lncRNA-422 were generated and prepared for RNA pull-down assays using the Pierce™ Magnetic RNA-Protein Pull-Down Kit (Thermo Fisher, Waltham, USA) according to the manufacturer's instructions. The antisense sequence of lncRNA-422 was transcribed with biotin RNA labelling mix and T7 RNA polymerase (Invitrogen, CA, USA), and then incubated with streptavidin-linked magnetic beads. The bead-RNA-protein complexes were washed, precipitated, and diluted in protein lysis buffer. Finally, the eluted proteins were subjected to protein identification by one-shot mass spectrometry or western blotting.

**Mass spectrometry analyses**

The raw data were converted using MaxQuant (https://www.maxquant.org/), while the proteins were identified using UniProt software (https://sparql.UniProt.org/). A fragment ion mass tolerance of 0.10 Da and a parent ion tolerance of 20.0 ppm were used. Scaffold software was used to confirm MS/MS-based peptide and protein recognitions. Only proteins with 1% protein FDR, at least two peptides per protein, 0.1% peptide FDR, and relevant sense sequences not found in the antisense control were considered. The identified proteins are listed in **Table S2**.

**RNA-binding protein immunoprecipitation (RIP) assay**

Colorectal cancer cells after different treatments were rinsed, fixed, and lysed in ice-cold lysis buffer. After centrifugation, magnetic beads binding rabbit SFPQ antibody (ab38148; Abcam, Shanghai, China) and nonspecific rabbit IgG antibody were prepared. After immunoprecipitation of the RNA-binding protein-RNA complex, RNA was precipitated and isolated to remove any genomic DNA contamination treated with DNase I from the EZ-Magna RIP Kit (Millipore, MA, USA) following the protocol provided by the manufacturer. Briefly, each sample was reverse-transcribed by quantitative mRNA analysis.

**Western blotting**

Cells were lysed in lysis buffer and extracted proteins were separated by 8%-10% SDS-PAGE and then transferred onto PVDF membranes (Millipore, MA, USA). After blocking membranes, they were incubated with primary antibodies against SFPQ (Abcam, 1:1,000), RBM14 (Abcam, 1:500), ALYREF (Abcam, 1:1,000), or internal control β-actin (Beyotime, 1:1,000) and then with horseradish peroxidase-conjugated secondary antibodies. Signals were detected and obtained by using the ECL system (Thermo Fisher Scientific, NY, USA).

**Cell proliferation assay and colony formation assay**

For cell proliferation assays, cells were cultured and quantified in 96-well plates overnight before experimental treatments. Cell Counting Kit-8 (CCK-8) assays (Dojindo, Kumamoto Prefecture, Japan) were used and incubated at 37°C for 2 h. Cells in the logarithmic growth phase were analysed using an EdU Assay Kit (RiboBio, C10310-2). After EdU labelling, paraformaldehyde fixation, Apollo staining, and DNA staining, images were taken under a fluorescence microscope. The absorbance at 450 nm was detected, and each experiment was recorded continuously for 3 days. For colony formation assays, cells were seeded and incubated in a 6-well plate. Until visible colonies formed in the complete growth medium, megascopic cell colonies were fixed, stained with crystal violet (Sigma-Aldrich, MO, USA), and photographed two weeks later.

**Flow cytometry assay**

For cell cycle analysis, transfected cells were gathered and fixed with 70% ethanol on ice for 1 h. Cell-cycle progression was measured using propidium iodide (PI) staining (BD Biosciences, NJ, USA). After discarding the supernatant, the cell pellet was resuspended in 0.5 ml PBS containing 20 μl of PI and incubated in a dark room for 30 min. For cell apoptosis analysis, cells were double-stained with 100 μl of 1 × binding buffer containing 5 μl of PI and 5 μl of Annexin‐V‐FITC. After incubation in a dark room for 15 min, cells were subjected to apoptosis analysis using FACScan (BD Biosciences, NJ, USA).

**Xenograft mouse model**

HCT116 cells expressing stable overexpression of lncRNA-422 were subcutaneously injected into the dorsal flank of the same athymic 5-week-old male BALB/c nude mice (n = 8 for each group, Shanghai SLAC Laboratory Animal Co. Ltd.). The subcutaneous tumor volume (V) = (a × b^2^)/2, and the tumor growth curve are depicted. Nude mice were sacrificed by carbon dioxide asphyxiation. All animal care guidelines were conducted following the National Institutes of Health’s Guide for the Care and Use of Laboratory Animals. All studies on animals were approved by the Institutional Animal Care and Use Committee of Nanjing Medical University (IACUC: 2005055).

**Immunohistochemical staining**

Paraffin-embedded tissue sections were dewaxed and rehydrated before antigen retrieval. Tissues were incubated with goat serum for 30 min at room temperature and then incubated with SFPQ (Abcam, 1:50), and HRP-conjugated anti-mouse IgG (Millipore, MA, USA), and measured by DAB (diaminobenzidine) according to the manufacturer’s instructions. For imaging, cells were DAPI-counterstained and recorded for evaluation by a digital system on a DMI3000 B microscope. The staining scores were determined and divided blindly by two inspectors.

**Statistical analysis**

All statistical analyses were coded with R software (version 3.5.1). All graphs were generated using GraphPad Prism Software (version 8.0). All expression data transformed by log2 are presented as the mean ± standard deviation (SD) or median with 95% confidence interval (95% CI). Differences in mean values between two groups were analysed by two-sided Student's *t*-test and one-way ANOVA. Statistical comparisons were performed using the Mann-Whitney test, Kruskal-Wallis test, or Pearson’s correlation analysis, as appropriate. Differences with *P* < 0.05 were considered statistically significant.

**References**

1. Lin MF, Jungreis I, Kellis M. PhyloCSF: a comparative genomics method to distinguish protein coding and non-coding regions. Bioinformatics (Oxford, England) 2011;27(13):i275-82.

2. Wang L, Park HJ, Dasari S, Wang S, Kocher JP, Li W. CPAT: Coding-Potential Assessment Tool using an alignment-free logistic regression model. Nucleic acids research 2013;41(6):e74.

3. Kong L, Zhang Y, Ye ZQ, et al. CPC: assess the protein-coding potential of transcripts using sequence features and support vector machine. Nucleic acids research 2007;35(Web Server issue):W345-9.
